# A 3D atlas of the dynamic and regional variation of pancreatic innervation in diabetes

**DOI:** 10.1126/sciadv.aaz9124

**Published:** 2020-10-09

**Authors:** Alexandra Alvarsson, Maria Jimenez-Gonzalez, Rosemary Li, Carolina Rosselot, Nikolaos Tzavaras, Zhuhao Wu, Andrew F. Stewart, Adolfo Garcia-Ocaña, Sarah A. Stanley

**Affiliations:** 1 Diabetes, Obesity, and Metabolism Institute, Division of Endocrinology, Diabetes and Bone Diseases, Icahn School of Medicine at Mount Sinai, New York, NY 10029, USA.; 2 The Microscopy CoRE and Advanced Bioimaging Center, Icahn School of Medicine at Mount Sinai, New York, NY 10029, USA.; 3 Department of Cell, Developmental & Regenerative Biology, The Icahn School of Medicine at Mount Sinai, New York, NY 10029, USA.; 4 Department of Neuroscience, Icahn School of Medicine at Mount Sinai, New York, NY 10029, USA.; 5 The Mindich Child Health and Development Institute, The Icahn School of Medicine at Mount Sinai, New York, NY 10029, USA.

## Abstract

Understanding the detailed anatomy of the endocrine pancreas, its innervation, and the remodeling that occurs in diabetes can provide new insights into metabolic disease. Using tissue clearing and whole-organ imaging, we identified the 3D associations between islets and innervation. This technique provided detailed quantification of α and β cell volumes and pancreatic nerve fibers, their distribution and heterogeneity in healthy tissue, canonical mouse models of diabetes, and samples from normal and diabetic human pancreata. Innervation was highly enriched in the mouse endocrine pancreas, with regional differences. Islet nerve density was increased in nonobese diabetic mice, in mice treated with streptozotocin, and in pancreata of human donors with type 2 diabetes. Nerve contacts with β cells were preserved in diabetic mice and humans. In summary, our whole-organ assessment allows comprehensive examination of islet characteristics and their innervation and reveals dynamic regulation of islet innervation in diabetes.

## Teaser

Three-dimensional mapping of pancreatic innervation shows rapid remodeling of islet innervation in mouse and human diabetes.

## INTRODUCTION

Insulin-producing β cells do not exist in isolation, and their environment has substantial effects on their architecture and function. In addition to the adjacent α, delta, ghrelin, pancreatic polypeptide, and other endocrine cells, the exocrine pancreas, vasculature, and innervation all modify β cell organization and insulin release (*1*). Islets are innervated by autonomic parasympathetic and sympathetic fibers, as well as by sensory fibers (*2*, *3*). Evidence from many studies over the past century has identified a critical role for neural signals in modulating insulin and glucagon release to regulate blood glucose (*4*). For example, anticipatory signals increase insulin release upon food consumption but before any changes in blood glucose, and neural signals suppress insulin and stimulate glucagon release to counteract hypoglycemia (*4*). Since central nervous system (CNS) and nerve stimulation studies demonstrate that neural signals can override the effects of circulating glucose (*5*, *6*), neural modulation is an attractive target for therapies to improve metabolic control.

Our current understanding of islets and their innervation largely relies on traditional histological techniques using immunolabeled structures in thin sections. These studies have provided a wealth of knowledge about islet structure at high resolution. However, pancreata are highly heterogeneous (*7*), with distinct regional embryological origins. Sections also lack landmarks to precisely and consistently identify the location of internal structures (*8*). Until now, laborious serial sectioning and reconstruction have been needed to deliver information about islet anatomy throughout the pancreas. In addition, thin filamentous structures, such as nerves, are difficult to quantify and trace over large volumes using this approach. Recent studies have applied confocal imaging of small pieces and thick sections of cleared pancreatic tissue to examine endocrine innervation (*9*–*14*). These have revealed dense nerve processes within both mouse and human islets. However, given the heterogeneity in the pancreas, there is a clear need for high-resolution, organ-wide imaging to accurately quantify and map regional variation and to assess the three-dimensional (3D) relationship between islets and their environment in health and disease.

Here, we used a tissue-clearing technique, iDISCO^+^ (*15*), to determine the 3D distribution of insulin-producing β cells, glucagon-producing α cells, and neurofilament 200 kDa (NF200)–positive innervation across the whole pancreas in healthy animals and in mouse models of diabetes. NF200 is a pan-neuronal marker expressed in sympathetic, sensory, and vagal neurons but, unlike other neural markers, is not expressed in pancreatic endocrine cells (*16*–*18*). NF200 is expressed in small and large myelinated and small unmyelinated fibers (*19*), so examining NF200^+^ fibers provides a comprehensive overview of pancreatic innervation. In addition, NF200 protein levels are altered by nerve damage and repair (*20*–*22*), so NF200 intensity may reflect remodeling of pancreatic nerves. Using whole-organ 3D imaging and analysis, we readily quantified β cell volume and provide detailed information about islet distribution and heterogeneity in mouse and human pancreatic tissue from healthy and diabetic donors. We quantified the dense endocrine innervation and its regional variation and demonstrated significant differences between innervated and noninnervated islets. Islet nerve density is significantly increased in diabetic nonobese diabetic (NOD) mice, with streptozotocin (STZ) treatment, and greater in pancreatic tissue from diabetic human donors. We systematically quantified intrapancreatic ganglia and nerve contacts with α and β cells to demonstrate that these are largely preserved in diabetes. These findings constitute a “3D atlas of pancreatic innervation” for pancreas and diabetes investigators examining pancreatic innervation, the regional heterogeneity in the healthy pancreas, and responses to metabolic disease. Our studies suggest that diabetes is associated with significant remodeling of neural inputs into islets and that neural contacts with endocrine cells are preserved in diabetes.

## RESULTS

### Islet distribution and insulin staining show regional differences in C57BL/6 mice

**Fig. 1:**
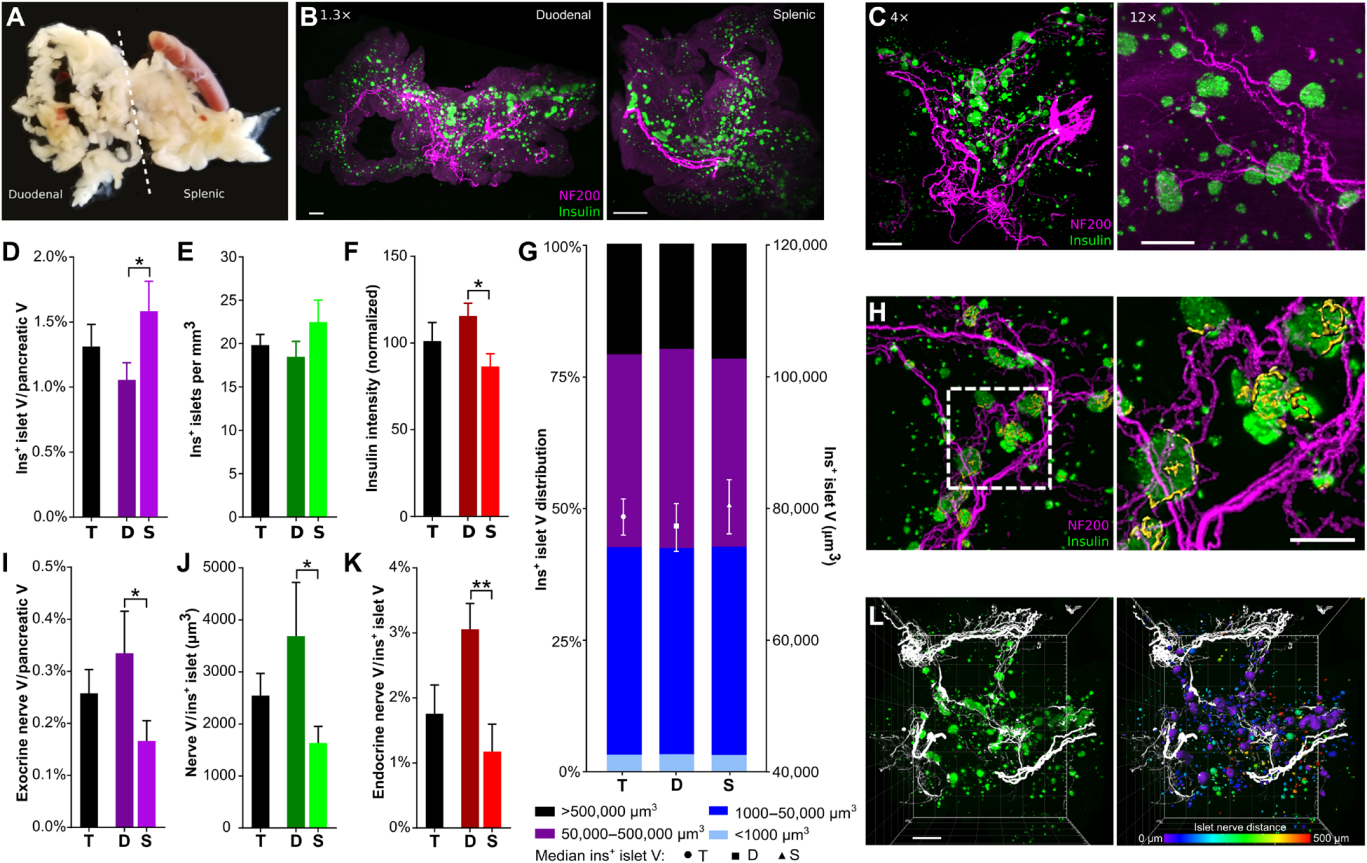
β cell distribution and pancreatic innervation in C57BL/6 mice. (**A**) Pancreatic dissection. Photo credit: A.A., Icahn School of Medicine at Mount Sinai. (**B**) Duodenal (left) and splenic (right) pancreas, maximum projection (1.3×). Scale bars, 500 μm. (**C**) Pancreata, maximum projection at 4× (left) and 12× (right). Scale bars, 500 and 200 μm. (**D**) β cell volume. (**E**) Insulin^+^ islets per cubic millimeter. (**F**) Insulin intensity (normalized to whole pancreas). (**G**) Insulin^+^ islet volume distribution (left axis) and median volume (right axis). Islets per group: 27,092/12,260/14,832. (**H**) 3D projection of insulin, NF200^+^ exocrine innervation, and NF200^+^ surfaces within insulin^+^ islets (yellow). (**I**) Exocrine nerve volume. (**J**) Endocrine nerve volume per insulin^+^ islet. (**K**) Endocrine nerve volume/islet volume. (**L**) Left: 3D model of pancreatic innervation (NF200, white) and insulin (green). Right: Distance transformation analysis with islet surfaces pseudocolored based on distance from the nearest nerve surface. Scale bar, 500 μm. Boxed area magnified in the right panel. Scale bar, 200 μm. Data are shown as means ± SEM or median ± 95% confidence interval as indicated. Analyses by unpaired *t* test, *P < 0.05 and **P < 0.01. T, total; D, duodenal; S, splenic. *N* = 7 (D to G) and *N* = 5 (I to K).

We applied tissue clearing and whole-organ 3D imaging to examine β cell mass, expressed as β cell volume, and islet number, as well as spatial distribution in whole pancreata from C57BL/6 mice (Fig. 1, A to C, and movies S1 and S2).

The total β cell volume made up 1.31 ± 0.17% of the total pancreatic volume (Fig. 1D), with a greater β cell volume in the splenic region. In line with previous reports (*23*, *24*), there were 3874 ± 264.2 islets per pancreas, with 1822 ± 230.4 in the duodenal and 2052 ± 129 in the splenic regions. Islet density (islet number per cubic millimeter) did not differ significantly across the pancreas (Fig. 1E). Insulin intensity showed significant regional variation with intensity in the duodenal pancreas being 25% greater than that in the splenic region (Fig. 1F).

We next examined islet distribution throughout the pancreas to determine whether there were regional differences in β cell volume per islet (Fig. 1G). Islets with β cell volumes between 1000 and 50,000 μm^3^ were the most abundant (39.29%), followed by islets in the 50,000 to 499,999 μm^3^ range (36.58%). Very large islets (>500,000 μm^3^) comprised 20% of the islet population, and insulin^+^ structures with volumes below 1000 μm^3^, consisting of five or fewer β cells, were the least abundant (3.13%).

### Islet innervation shows regional variation in C57BL/6 mice

There are reported differences in the origins of nerves innervating the duodenal and splenic pancreas (*25*). Therefore, we hypothesized that there may be regional variations in pancreatic innervation. Thus, we next analyzed the 3D distribution of the pan-neuronal marker NF200 in the healthy mouse pancreas to determine regional variations and relationship to islets (Fig. 1H and movies S3 to S5).

The exocrine nerve volume was 40% greater in the duodenal pancreas compared with the splenic region (Fig. 1I). Pancreatic islets were highly innervated compared to exocrine tissue, with an endocrine nerve density over sixfold greater than the exocrine nerve density. In addition, there was significant regional variation in islet innervation. Nerve volume per islet in the duodenal region was almost double that in the splenic region (Fig. 1J). This difference was more pronounced when the endocrine nerve volume was corrected for β cell volume (Fig. 1K). These findings are consistent with marked regional variation in the density of islet innervation.

### Large islets are innervated by NF200 fibers

**Fig. 2:**
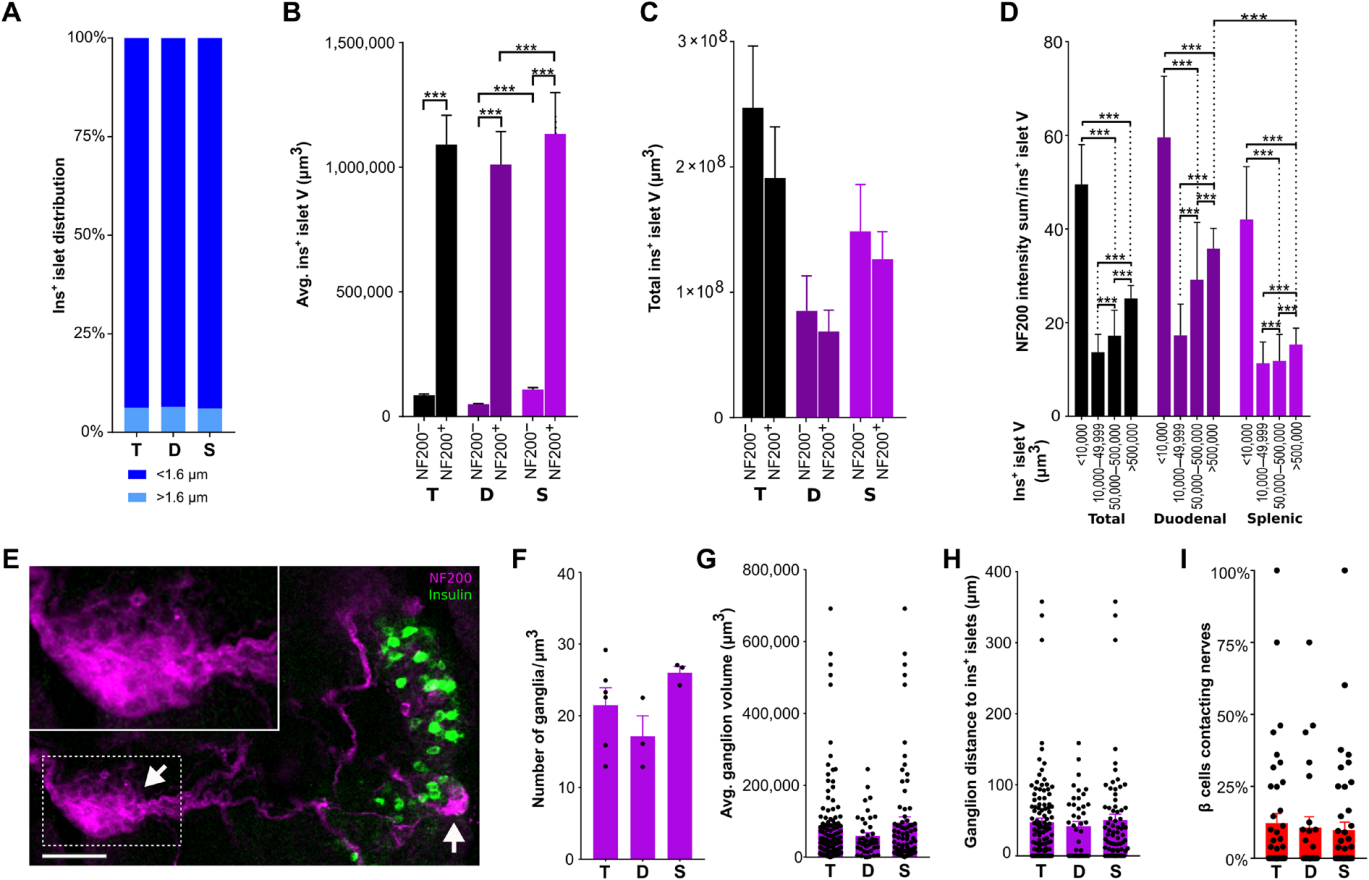
Interactions between β cells and innervation in C57BL/6 mice. (**A**) Distribution of insulin^+^ islets (<1.6 and >1.6 μm from the nearest nerve). Islets per group: 25,310/10,030/15,280. (**B**) Mean insulin^+^ islet volume ± NF200^+^ innervation; islets per group: 11,869/929/4690/325/7179/604. (**C**) Total insulin^+^ islet volume ± NF200^+^ innervation. (**D**) NF200 intensity sum normalized for insulin^+^ islet volume; islets per group: 5174/4530/2264/687/2196/1788/701/330/2978/2742/1563/357. (**E**) Intrapancreatic ganglia (NF200, magenta) and β cells (insulin, green). Arrows mark ganglia. Scale bar, 50 μm. (**F**) NF200^+^ intrapancreatic ganglia per cubic millimeter. (**G**) Intrapancreatic ganglia volume. Ganglia per group: 123/43/80. (**H**) Distance between intrapancreatic ganglia and insulin^+^ islets. Ganglia per group: 123/43/80. (**I**) β cells contacting nerves per islet. Islets per group: 69/40/29. Data are shown as means ± SEM or median ± 95% confidence interval as indicated. Analyses by Kruskal-Wallis test with Dunn’s test (B to D) or unpaired *t* test (F to I), ***P < 0.001. T, total; D, duodenal; S, splenic. *N* = 5 (A to D), *N* = 3 (F to H), and *N* = 4 (I).

The proximity of nerves and endocrine cells may have important biological consequences. Autonomic neurotransmission occurs over 1 to 2 μm (*26*), but endocrine and immune cells may influence nerve growth, repair, and function over longer ranges (*27*, *28*). As a result, we examined the proportion of islets in contact with NF200^+^ fibers and the distance of each islet from the closest NF200^+^ fiber (Fig. 1L and movie S6). Only 6.1% of islets contained or were in contact with NF200^+^ fibers, with no significant difference between duodenal and splenic regions (Fig. 2A). The proportion of innervated (NF200^+^) islets increased with islet volume (fig. S1C). Most islets were within 250 μm of an NF200^+^ fiber, and islets in the duodenal pancreas were significantly closer to nerves than those in the splenic pancreas (fig. S1A).

To test the hypothesis that innervated islets differ from those without innervation, we then analyzed islet volume based on whether islets were innervated by NF200^+^ fibers, hypothesizing that neural signals may play a role in determining islet size. NF200-innervated islets were 10-fold larger than islets without NF200 innervation (Fig. 2B and fig. S1B), and as a result, innervated islets made up 43% of the total β cell volume in the pancreas (Fig. 2C). Both innervated and noninnervated islets in the splenic region were larger than those in the duodenal pancreas (Fig. 2B).

Next, we analyzed the intensity of NF200^+^ immunostaining within each islet. NF200 protein levels are associated with structural stability of nerves and increased conduction velocity, so NF200^+^ immunostaining intensity may have functional correlates (*29*, *30*). While the largest islets were more likely to be innervated, the intensity of NF200^+^ immunostaining was twofold greater in the smallest islets compared to the largest islets and greater in subpopulations of duodenal islets (Fig. 2D).

These data demonstrate that innervated islets are a small fraction of the total islet number but are significantly larger than islets without NF200 innervation and form a substantial portion of the total β cell volume. These findings suggest the potential involvement of NF200^+^ nerves in islet development and β cell growth.

### Intrapancreatic ganglia and nerve/β cell contacts are sparse

Intrapancreatic ganglia integrate inputs from the sympathetic and parasympathetic nervous systems and provide significant islet innervation (*31*). Regional differences in ganglia size in the pancreas have been reported (*32*). Intrapancreatic ganglia are sparse (21.5 ± 2.5 ganglia/mm^3^; Fig. 2, E and F), with an average volume of 83,467 ± 10,646 μm^3^ (Fig. 2G) and located close to islets (47.3 ± 5.7 μm; Fig. 2H). There were no significant regional differences in ganglia density, size, or location.

To assess whether islet innervation could directly influence endocrine cell function through neural signals, we quantified the number of β cells contacting NF200^+^ nerves. Only 9.4 ± 2.2% of β cells contacted NF200^+^ nerves (Fig. 2I) with no regional difference. As expected, a larger number of β cells contacted nerves in large islets compared to small islets (fig. S1D), but the proportion of β cells contacting NF200^+^ nerves did not differ with islet size (fig. S1E). In aggregate, these data provide a comprehensive 3D atlas of the anatomy and NF200^+^ innervation of the entire mouse endocrine and exocrine pancreas that can be used as a benchmark to assess the effects of specific pancreatic innervation during development and in disease.

### Regional variation in islet characteristics in NOD mice

**Fig. 3:**
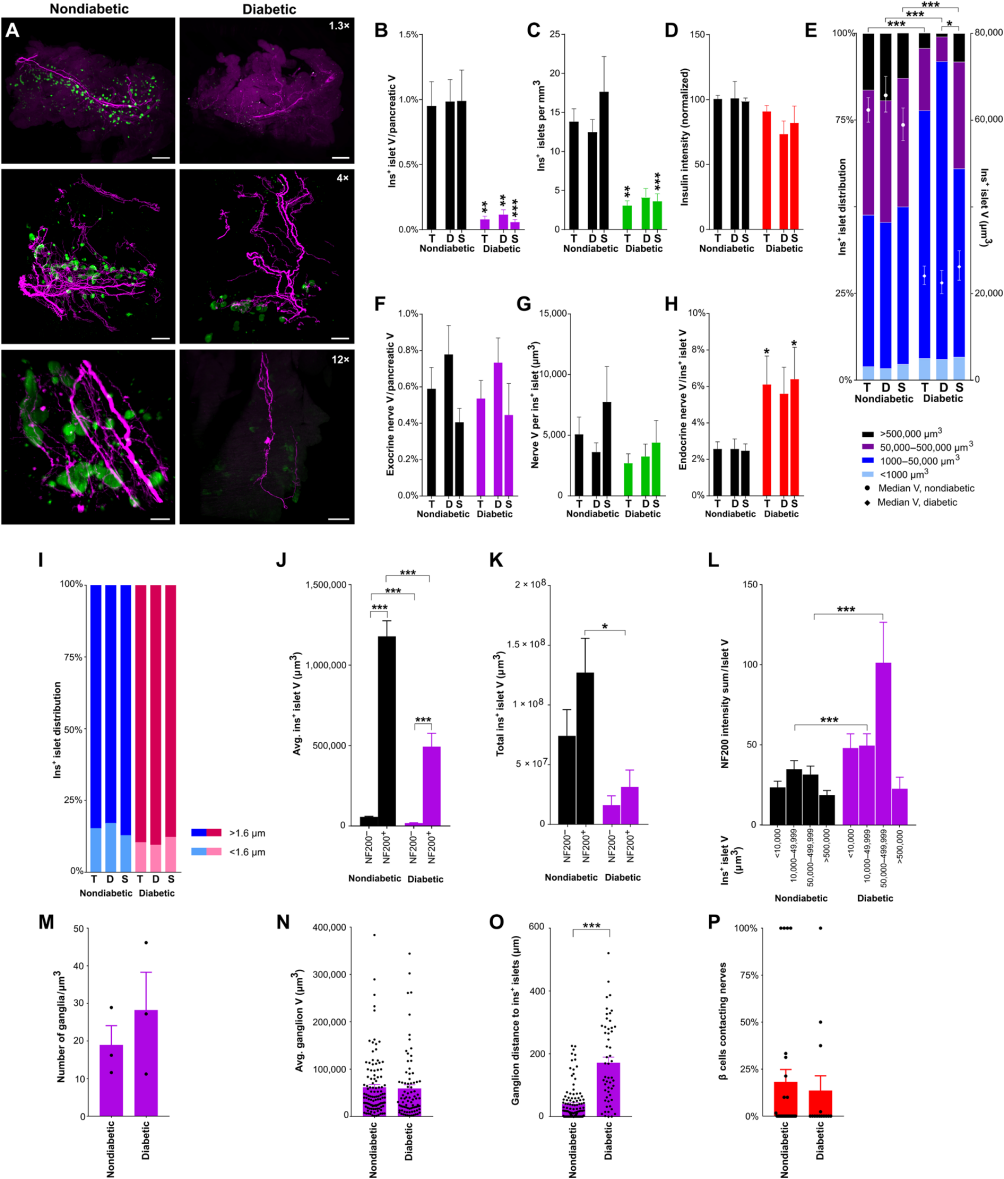
β cell distribution and innervation in nondiabetic and diabetic nonobese diabetic (NOD) mice. (**A**) Pancreata from nondiabetic and diabetic NOD mice [maximum projection at 1.3× (top), 4× (middle), and 12× (bottom); scale bars, 2000, 500, and 200 μm]. (**B**) β cell volume. (**C**) Insulin^+^ islets per cubic millimeter of pancreatic tissue. (**D**) Insulin intensity (normalized against total pancreas, nondiabetic). (**E**) Insulin^+^ islet volume distribution (left axis) and median volume (right axis). Islets per group: 11,404/6285/5119/4057/2203/1854. (**F**) Exocrine nerve volume. (**G**) Endocrine nerve volume per insulin^+^ islet. (**H**) Endocrine nerve volume by islet volume. (**I**) Distribution of insulin^+^ islets located <1.6 and >1.6 μm from the nearest nerve. (**J**) Mean insulin^+^ islet volume ± NF200^+^ innervation. Islets per group: 8815/857/4296/436. (**K**) Total insulin^+^ islet volume ± NF200^+^ innervation. (**L**) NF200 intensity sum normalized for insulin^+^ islet volume. Islets per group: 4941/3341/1209/383/2862/1586/189/73. (**M**) Intrapancreatic ganglia per cubic millimeter. (**N**) Intrapancreatic ganglia volume. Ganglia per group: 112/82. (**O**) Distance between intrapancreatic ganglia and insulin^+^ islets. Ganglia per group: 111/54. (**P**) β cells contacting nerves per islet. Islets per group: 28/14. Data are shown as means ± SEM or median ± 95% confidence interval where indicated. Analyses by one-way ANOVA with Tukey’s test (B to D and F to G), Kruskal-Wallis with Dunn’s test (H and J to L), or unpaired *t* test between diabetic and nondiabetic groups (H). *P < 0.05, **P < 0.01, and ***P < 0.001. T, total; D, duodenal; S, splenic. *N* = 7 nondiabetic and *N* = 7 diabetic (B to E, P); *N* = 8 nondiabetic and *N* = 7 diabetic (F to L); *N* = 6 nondiabetic and *N* = 6 diabetic (M to O).

The 3D relationships between islets and innervation across the whole endocrine pancreas are largely unknown in diabetes. Hence, we determined how pancreatic anatomy and β cell innervation were affected in a mouse model of type 1 diabetes (T1D). NOD mice provide a model of diabetes with autoimmune β cell destruction and spontaneous T1D development. We examined the 3D structure of NF200^+^ innervation and islets in nondiabetic NOD mice (average nonfasting blood glucose, 115 ± 4 mg/dl) and diabetic NOD mice (average nonfasting blood glucose, 495 ± 62 mg/dl; Fig. 3A and movies S7 and S8).

Across the whole pancreas, islet density and β cell volume in female nondiabetic NOD mice were similar to that seen in male C57BL/6 mice (Figs. 1, D and E, and 3, A to C). In female diabetic NOD mice, the β cell volume was significantly lower across the whole pancreas, reduced to 10% of the volume in nondiabetic NOD mice in both splenic and duodenal regions (Fig. 3B). The islet number was also significantly reduced in diabetic NOD mice, particularly in the splenic, but not duodenal pancreas (Fig. 3C). However, the intensity of insulin immunostaining was preserved in the remaining islets that were detected in diabetic NOD mice (Fig. 3D). There was a significant inverse correlation between blood glucose levels and both islet number and β cell volume (fig. S2A).

The volume distribution of insulin^+^ islets in nondiabetic NOD mice was also comparable to C57BL/6 mice (Fig. 3E). However, islet volume distribution was significantly shifted in diabetic NOD mice, with marked loss of larger islets. Insulin^+^ islets over 50,000 μm^3^ were reduced by more than half, and the median islet volume decreased by more than 50%. The loss of large islets was particularly notable in the duodenal pancreas (Fig. 3E).

Together, these data demonstrate marked decreases in insulin^+^ islet number and volume and marked alterations in islet volume distribution in diabetic compared to nondiabetic NOD mice, particularly in the duodenal pancreas. Our data also suggest that the remaining islets in diabetic NOD mice maintained their insulin content.

### Islet innervation is significantly increased in diabetic NOD mice

Previous studies have reported alterations in pancreatic innervation in mouse models of diabetes (*13*, *33*–*36*). Therefore, we examined pancreatic innervation in NOD mice to determine effects on nerve density in the different regions of the pancreas (movies S9 and S10).

Nerve density in insulin^+^ islets was increased more than twofold in diabetic NOD mice (Fig. 3, G and H), particularly in the splenic pancreas. Islet nerve density in the splenic pancreas positively correlated with blood glucose (fig. S2C). The regional differences in endocrine nerve density observed in C57BL/6 mice were absent in nondiabetic NOD mice. There was no difference in exocrine nerve density between nondiabetic and diabetic NOD mice and no correlation with blood glucose (Fig. 3F and fig. S2B).

Previous studies suggest that neural signals contribute to β cell survival (*37*), so increased islet innervation could result from differences in the susceptibility of innervated and noninnervated islets to immune destruction. To test this, we examined the proportion of NF200^+^ islets (islets containing or in contact with NF200^+^ fibers) in NOD mice. We did not see any significant change in the proportion of NF200^+^ islets (14.6 versus 9.8% islets in nondiabetic and diabetic NOD mice, respectively; Fig. 3I). However, the proportion of NF200^+^ islets was increased in a subset of islets with volumes between 50,000 and 500,000 μm^3^ in diabetic NOD mice (fig. S2F). The median distance between islets and nerves was similar in diabetic and nondiabetic NOD mice for the total pancreas but significantly reduced in the splenic pancreas (fig. S2D).

In keeping with the results in C57BL/6 mice, NF200^+^ islets were significantly larger than NF200^−^ islets in both diabetic and nondiabetic NOD mice (Fig. 3J), although, as expected, the average volume of both NF200^−^ and NF200^+^ islets decreased in diabetic NOD mice. Innervated insulin^+^ islets remained 60% of the total β cell volume in both diabetic and nondiabetic NOD mice (Fig. 3K).

In published studies, the intensity of NF200 immunostaining decreases with nerve damage and increases in nerve regeneration (*20*, *22*). To indirectly assess the effects of autoimmune diabetes on nerve integrity in islets, we examined the intensity of NF200 immunostaining in diabetic and nondiabetic NOD mice and found that the intensity of NF200 immunostaining was significantly increased in islets from diabetic NOD mice (Fig. 3L).

### Intrapancreatic ganglia and nerve/β cell contacts are maintained in diabetic NOD mice

We next examined intrapancreatic ganglia to determine whether autoimmune diabetes altered their distribution or size. There was no significant difference in intrapancreatic ganglia density (18.9 ± 5.2 versus 28.2 ± 10.1 ganglia/mm^3^, nondiabetic versus diabetic NOD mice, respectively; Fig. 3M) or volume (61,779 ± 5961 versus 59,348 ± 6977 μm^3^, nondiabetic versus diabetic NOD mice, respectively; Fig. 3N), but the distance between intrapancreatic ganglia and islets increased fourfold in diabetic NOD mice (40 ± 5.3 versus 171.7 ± 17.6 μm, nondiabetic versus diabetic, respectively; Fig. 3O).

Next, we examined the proportion of β cells in contact with NF200^+^ fibers in nondiabetic and diabetic NOD mice. Despite a significant increase in islet nerve density, there was no significant change in the proportion of β cells contacting nerves in diabetic NOD mice (Fig. 3P).

### α cell innervation is increased in diabetic NOD mice

**Fig. 4:**
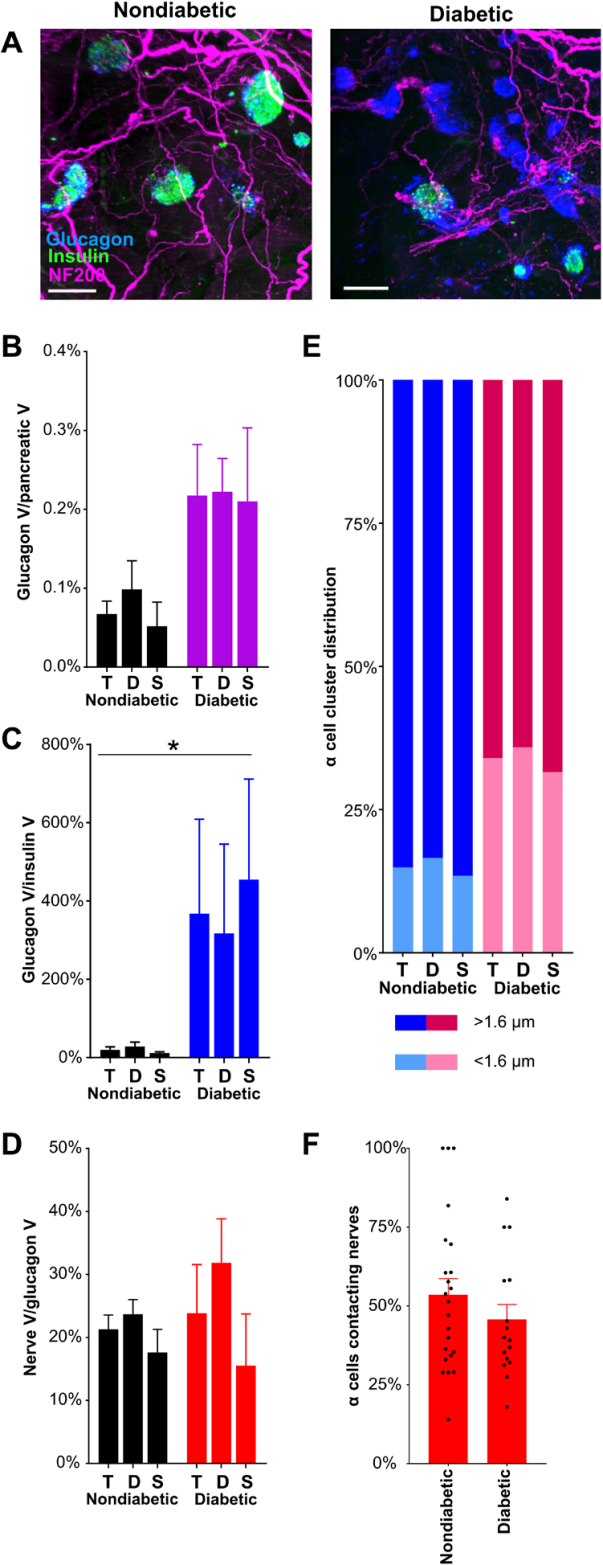
α cell distribution and innervation in nondiabetic and diabetic NOD mice. (**A**) Maximum projections of light-sheet images of pancreatic samples from nondiabetic and diabetic NOD mice stained for insulin (green), NF200 (magenta), and glucagon (blue) and imaged at ×4 magnification. Scale bars, 200 μm. (**B**) α cell volume corrected for pancreatic volume in NOD mice. (**C**) Glucagon^+^ α cell volume as a percentage of insulin^+^ β cell volume in NOD mice. (**D**) NF200^+^ nerve volume within glucagon^+^ α cell clusters in NOD mice. (**E**) Glucagon^+^ α cell cluster volume (left axis) and median nerve distance (right axis) in NOD mice. (**F**) Percentage of α cells contacting nerves per islet. Number of islets: 23/16. Data are shown as mean ± SEM or as median ± 95% confidence interval where indicated. Analyses by one-way ANOVA with Tukey’s test (D) or Kruskal-Wallis with Dunn’s test (B, C, and E). *P < 0.05. T, total; D, duodenal; S, splenic. *N* = 3 nondiabetic and *N* = 3 diabetic NOD mice.

Autoimmune cell destruction principally affects β cells in NOD mice resulting in islets composed primarily of glucagon^+^ α cells. The changes in α cell innervation in mouse models of diabetes are largely unknown. In diabetic NOD mice, glucagon staining is clearly present, but glucagon^+^ cells from a single islet may form several clusters rather than a clearly defined, single islet (Fig. 4, A and B). As previously reported (*38*), the ratio of glucagon to insulin volume (Fig. 4C) was significantly increased in diabetic NOD mice (movies S11 and S12). In nondiabetic NOD mice, NF200 nerve density in α cell clusters was markedly higher than nerve density in insulin^+^ islets. Nerve density in diabetic NOD mice was unchanged (Fig. 4D). The proportion of innervated α cell clusters was similar to that of innervated insulin^+^ islets in nondiabetic NOD mice and increased twofold in diabetic NOD mice (Fig. 4E). In keeping with increased NF200 nerve density in α cell clusters of nondiabetic NOD mice, the proportion of α cells contacting NF200^+^ fibers was more than fivefold higher than β cells contacting NF200^+^ fibers in nondiabetic NOD mice. However, the proportion of α cell nerve contacts did not change in diabetic mice (Fig. 4F).

In summary, insulin^+^ islet nerve density and NF200 immunostaining are increased in the surviving insulin^+^ islets of diabetic NOD mice, and β cell contacts with NF200^+^ fibers are preserved. α cell nerve density and α cell contacts with NF200^+^ fibers are greater than contacts with β cells, and α cell nerve density also increases in diabetic NOD mice.

### Multiple low-dose STZ treatment reduces islet volume and intensity of insulin immunostaining

**Fig. 5:**
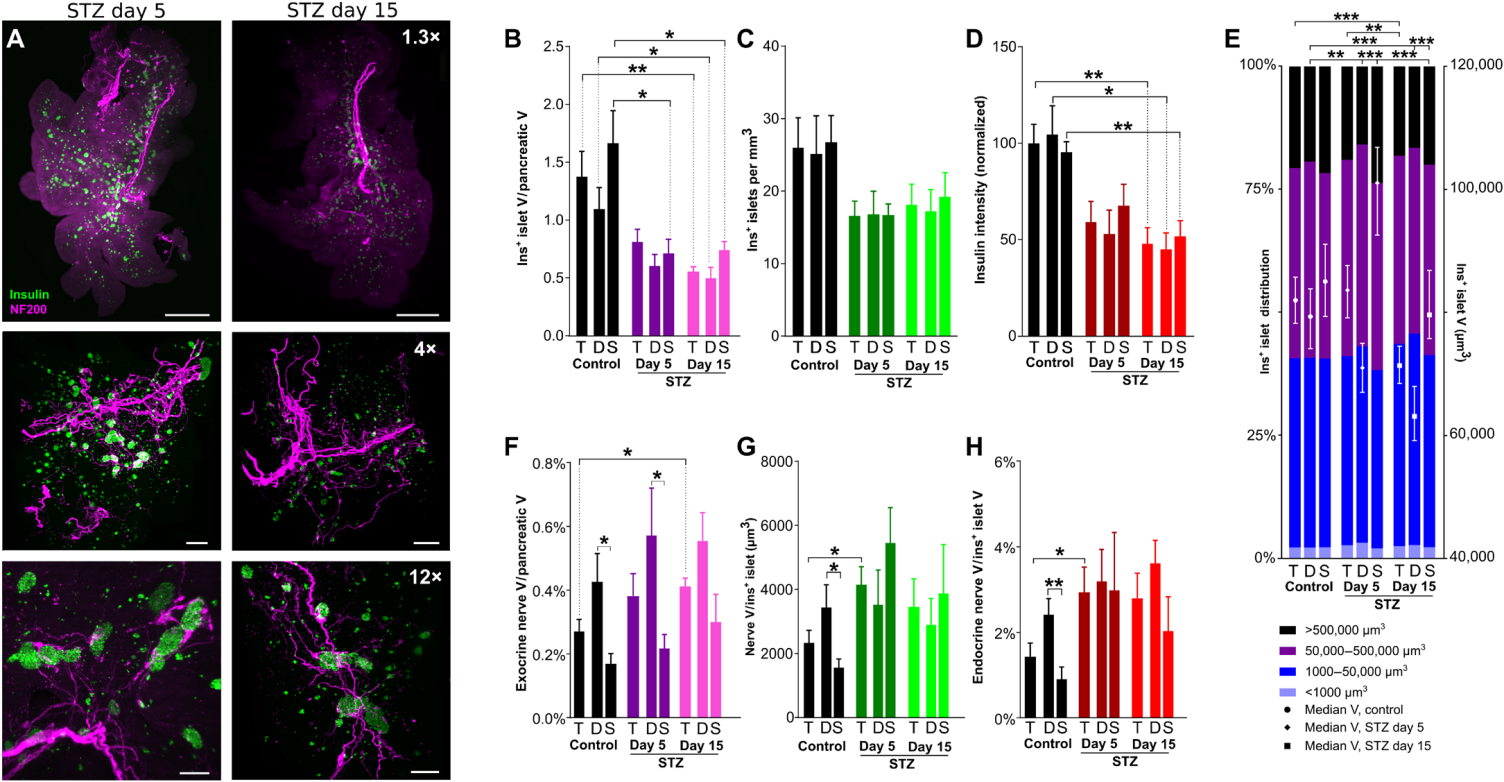
β cell distribution and pancreatic innervation with development of diabetes in STZ-treated mice. (**A**) Pancreata at days 5 (left) and 15 (right) after STZ treatment, maximum projections at 1.3× (top), 4× (middle), and 12× (bottom). Scale bars: 1000, 500, and 200 μm. (**B**) β cell volume. (**C**) Insulin^+^ islets per cubic millimeter. (**D**) Insulin intensity (normalized against total pancreas, control). (**E**) Insulin^+^ islet volume distribution (left axis) and median volume (right axis). Islets per group: 10,479/4682/5797/10,091/5162/4929/14,380/7543/6837. (**F**) Exocrine nerve volume. (**G**) Endocrine nerve volume per insulin^+^ islet. (**H**) Endocrine nerve by islet volume. Data are shown as mean ± SEM or as median ± 95% confidence interval where indicated. Analyses by one-way ANOVA with Tukey’s test for comparison between control, STZ day 5, and STZ day 15, and unpaired *t* test for comparison between duodenal and splenic pancreas (B to D and F to H) or Kruskal-Wallis with Dunn’s test (E); *P < 0.05, **P < 0.01, and ***P < 0.001. T, total; D, duodenal; S, splenic. *N* = 5 control, *N* = 6 STZ day 5, and *N* = 7 STZ day 15 (B to E); *N* = 6 control, *N* = 5 STZ day 5, and *N* = 5 STZ day 15 (F to H).

On the basis of our findings in NOD mice, we hypothesized that nerve density may progressively increase in surviving islets during the development of diabetes. To test this hypothesis, we examined the time course of changes in insulin^+^ islets and pancreatic nerves in mice with STZ-induced diabetes, as well as in age- and sex-matched C57BL/6 mice. Diabetes secondary to multiple low-dose STZ treatment is likely induced by both direct β cell toxicity and islet inflammation. Therefore, using a standard 5-day low-dose STZ model, we examined NF200, insulin, and glucagon staining in mice sacrificed 5 and 15 days after completion of STZ treatment (nonfasting blood glucose: 259 ± 18 and 430 ± 17 mg/dl, respectively) and compared these to untreated littermate controls (nonfasting blood glucose: 123 ± 9 mg/dl; Fig. 5A and movies S13 and S14).

First, we analyzed islet number and total β cell volume to determine the time course and effects of STZ treatment, hypothesizing that STZ may differentially affect these parameters in different pancreatic regions. As expected in this model, total β cell volume was reduced to 40% of control, and intensity of insulin immunostaining was decreased by 50% with STZ treatment (Fig. 5, B to D). Islet number and β cell volume negatively correlated with blood glucose in STZ-induced diabetes (fig. S3A). STZ treatment did not significantly alter the distribution of β cell volumes throughout the pancreas, suggesting that its effects were uniform across islets of all sizes (Fig. 5E). However, there was a significant decrease in the individual islet volume, first in the duodenal pancreas at day 5 and later at day 15 in both the duodenal and splenic regions. These findings demonstrate that STZ treatment progressively reduces total β cell volume, intensity of insulin immunostaining, and the volume of individual islets, with significant differences in the time course and extent of these changes between duodenal and splenic pancreas.

### STZ treatment increases endocrine innervation

We next examined pancreatic innervation in STZ-treated mice to determine the time course and regional distribution of effects on nerve density (movies S15 and S16). Nerve density in the exocrine pancreas was significantly increased 15 days after STZ treatment (Fig. 5F and fig. S3B). STZ treatment significantly increased islet innervation and islet nerve density by twofold on day 5 (Fig. 5, G and H). Islet nerve density was significantly correlated with blood glucose (fig. S3A).

**Fig. 6:**
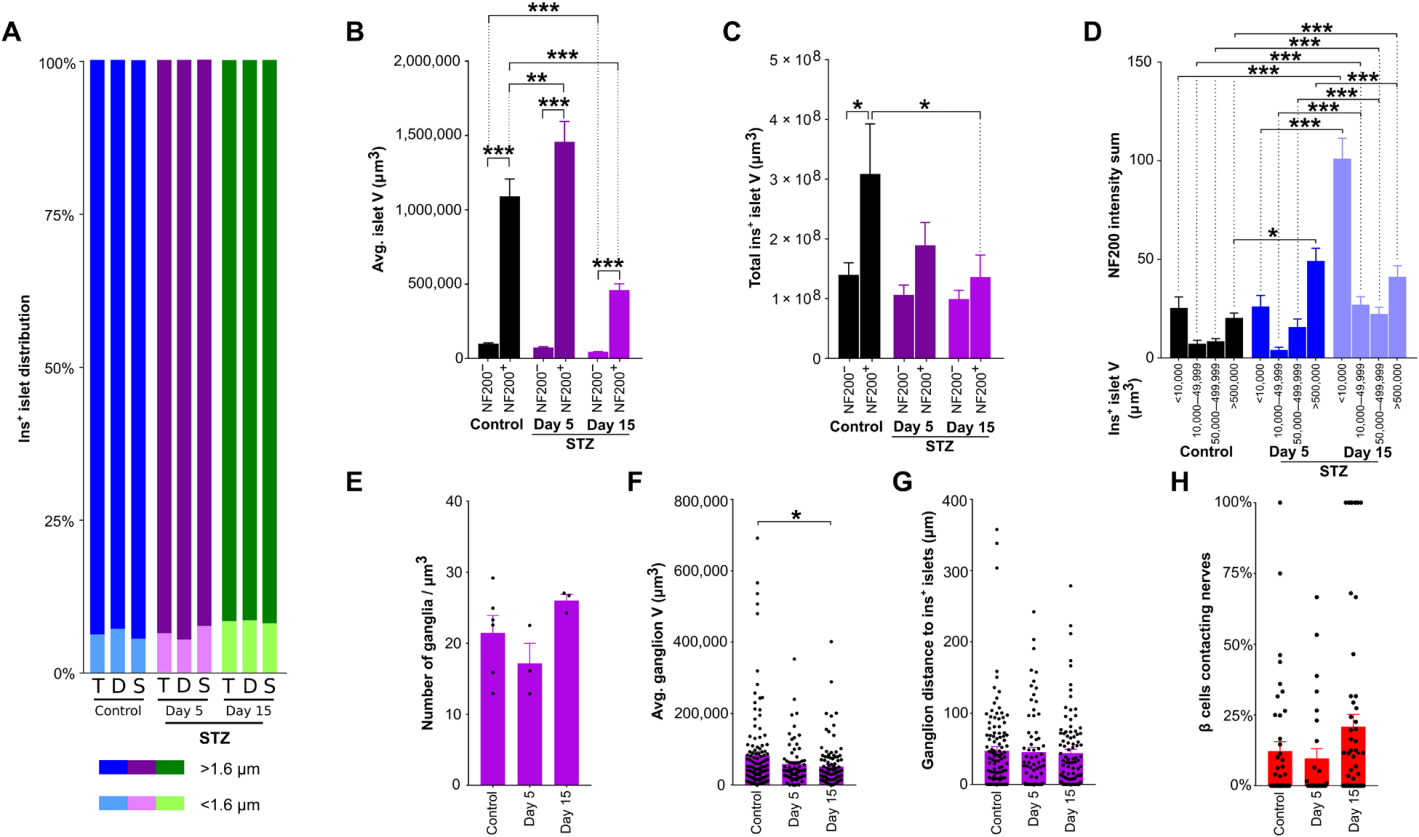
Interactions between β cells and innervation with development of diabetes in STZ-treated mice. (**A**) Distribution of insulin^+^ islets located <1.6 and >1.6 μm from the nearest nerve. (**B**) Mean volume for insulin^+^ islets ± NF200^+^ innervation. Islets per group: 10,199/929/5300/366/9837/1022. (**C**) Total volume for insulin^+^ islets ± NF200^+^ innervation. (**D**) Intensity of NF200 immunolabeling normalized for insulin^+^ islet volume. Islets per group: 3013/2762/1837/605/2842/1791/1057/336/5800/3274/1500/386. (**E**) Ganglia per cubic millimeter. (**F**) Volume of intrapancreatic ganglia. Ganglia per group: 114/73/97. (**G**) Distance between intrapancreatic ganglia and insulin^+^ islets. Ganglia per group: 114/73/97. (**H**) Percentage of β cells contacting nerves per islet. Islets per group: 69/28/69. Data are shown as means ± SEM or as median ± 95% confidence interval where indicated. Analyses by Kruskal-Wallis with Dunn’s test (B to D) for comparison between control, STZ day 5, and STZ day 15, and unpaired *t* test for comparison between duodenal and splenic pancreas (E to H). *P < 0.05, **P < 0.01, and ***P < 0.001. T, total; D, duodenal; S, splenic. *N* = 6 control, *N* = 5 STZ day 5, and *N* = 5 STZ day 15 (A to D); *N* = 6 control, *N* = 3 STZ day 5, and *N* = 3 STZ day 15 (E to H).

To test the hypothesis that neural signals may play a role in β cell preservation, we assessed whether STZ treatment had differential effects on islets based on whether they contained NF200^+^ nerves or not. STZ treatment led to a progressive increase in the proportion of NF200^+^ islets across the duodenal and splenic pancreas (Fig. 6A) and all islet sizes (fig. S3F) but did not reach significance (*P* = 0.14). STZ treatment significantly reduced the distance between insulin^+^ islets and NF200^+^ fibers on day 5, primarily in the splenic pancreas (fig. S3D). In both control and STZ-treated mice, innervated islets are significantly larger than noninnervated islets but decline in volume with STZ treatment (Fig. 6B). The total volume of innervated islets, but not of noninnervated islets, significantly decreased with STZ treatment (Fig. 6C) but remained 54% of the remaining total β cell volume.

To determine whether STZ-induced diabetes modified the expression of NF200, we assessed changes in intensity of NF200 immunostaining in relation to insulin^+^ islet volume and time after treatment (Fig. 6D). The intensity of NF200 immunostaining (corrected for β cell volume) was significantly increased in the largest islets (>500,000 μm^3^) 5 days after STZ treatment and by twofold to fourfold in all islets at 15 days after STZ treatment. These findings demonstrate that STZ treatment increases exocrine and endocrine nerve density and NF200 expression, results that are in keeping with increased nerve growth.

### Intrapancreatic ganglia and nerve/β cell contacts are maintained in STZ-treated mice

We next examined intrapancreatic ganglia in mice treated with STZ to determine whether β cell destruction changed their density or size. While STZ treatment did not change intrapancreatic ganglion density or distance from the islet, there was a 30% decrease in ganglion volume 15 days after STZ treatment (Fig. 6, E to G). Similar to our findings in NOD mice, although islet nerve density increased with STZ treatment, the proportion of β cells contacting NF200^+^ fibers did not change significantly (Fig. 6H).

### α cell nerve density is increased in STZ-treated mice

**Fig. 7:**
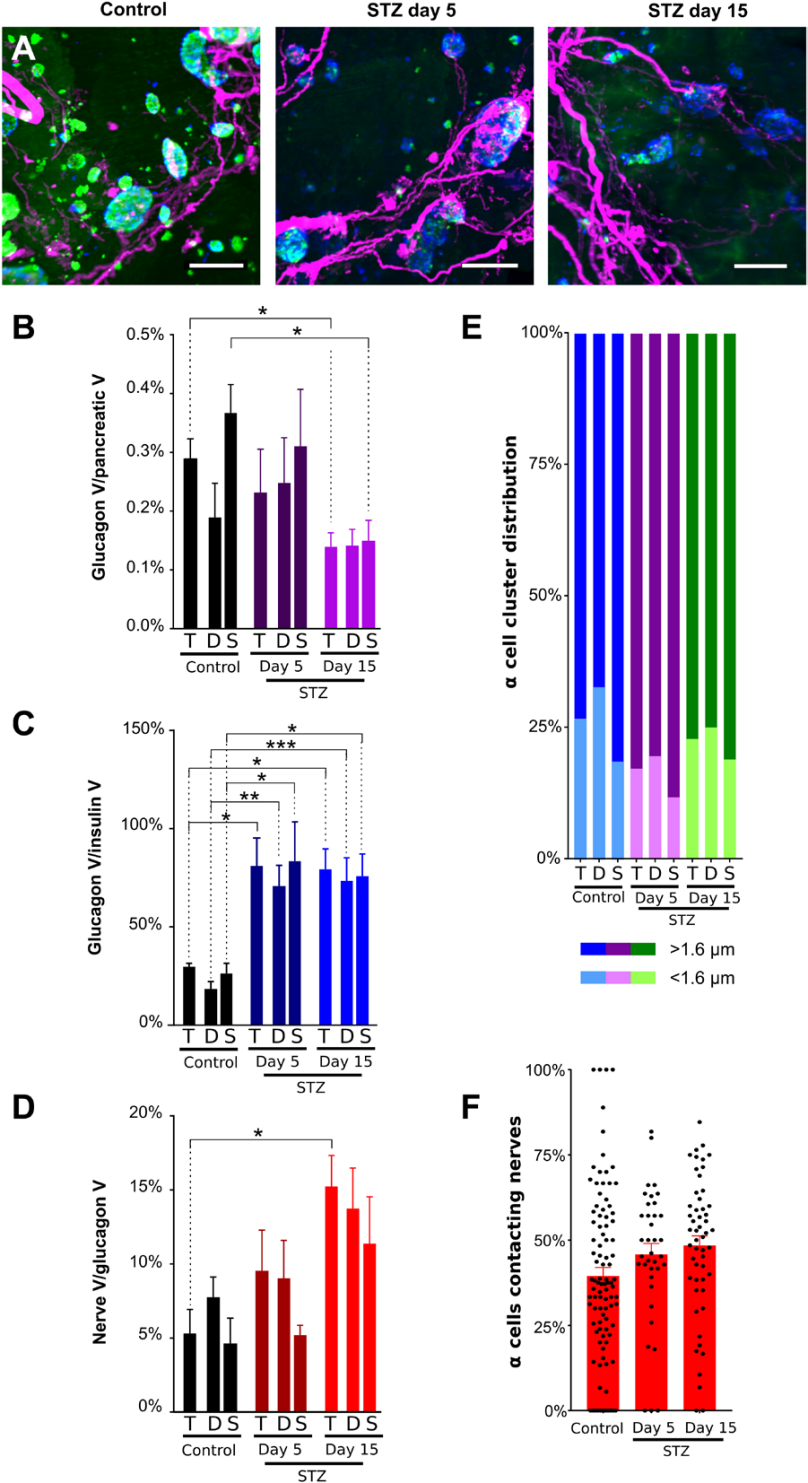
α cell distribution and innervation with development of diabetes in STZ-treated mice. (**A**) Pancreata at days 5 (left) and 15 (right) after STZ treatment. Insulin, green; NF200, magenta; glucagon, blue. Imaged at ×4 magnification. Scale bars, 200 μm. (**B**) Quantification of α cell volume corrected for pancreatic volume in STZ-treated mice. (**C**) Quantification of glucagon^+^ α cell volume as a percentage of insulin^+^ β cell volume in STZ-treated mice. (**D**) Quantification of NF200^+^ nerve volume within glucagon^+^ α cell clusters in STZ-treated mice. (**E**) Glucagon^+^ α cell cluster volume (left axis) and median nerve distance (right axis) in STZ-treated mice. (**F**) Percentage of α cells contacting nerves per islet. Islet number: 98/37/53. Data are shown as means ± SEM or as median ± 95% confidence interval where indicated. Analyses by one-way ANOVA with Tukey’s test (C to D) or Kruskal-Wallis with Dunn’s test (B and E). *P < 0.05, **P < 0.01, and ***P < 0.001. T, total; D, duodenal; S, splenic. *N* = 6 control, *N* = 4 STZ day 5, and *N* = 6 STZ day 15.

We next assessed α cell volume and nerve density in α cell clusters in STZ-treated mice. STZ treatment increased the ratio of glucagon^+^ to insulin^+^ cell volume, but total glucagon^+^ cell volume was reduced after 15 days (Fig. 7, A to C, and movie S17). NF200 nerve density in α cell clusters was significantly increased in STZ-treated mice (Fig. 7D), but the proportion of innervated α cell clusters did not change (Fig. 7E). Similarly, the proportion of α cells contacting NF200^+^ fibers was not significantly altered by STZ treatment (Fig. 7F).

In summary, 3D representation faithfully represents the progressive reduction in islet number, β cell volume, and intensity of insulin immunostaining in response to STZ treatment. Further, STZ treatment increases insulin^+^ islet nerve density, the proportion of innervated islets, and intensity of NF200 immunostaining. α cell nerve density is increased with STZ treatment, but the proportion of α and β cells that are in contact with NF200^+^ fibers is not significantly altered in STZ-treated mice.

### Islet and pancreatic nerve characteristics in optically cleared pancreatic tissue from nondiabetic and diabetic human donors

**Fig. 8:**
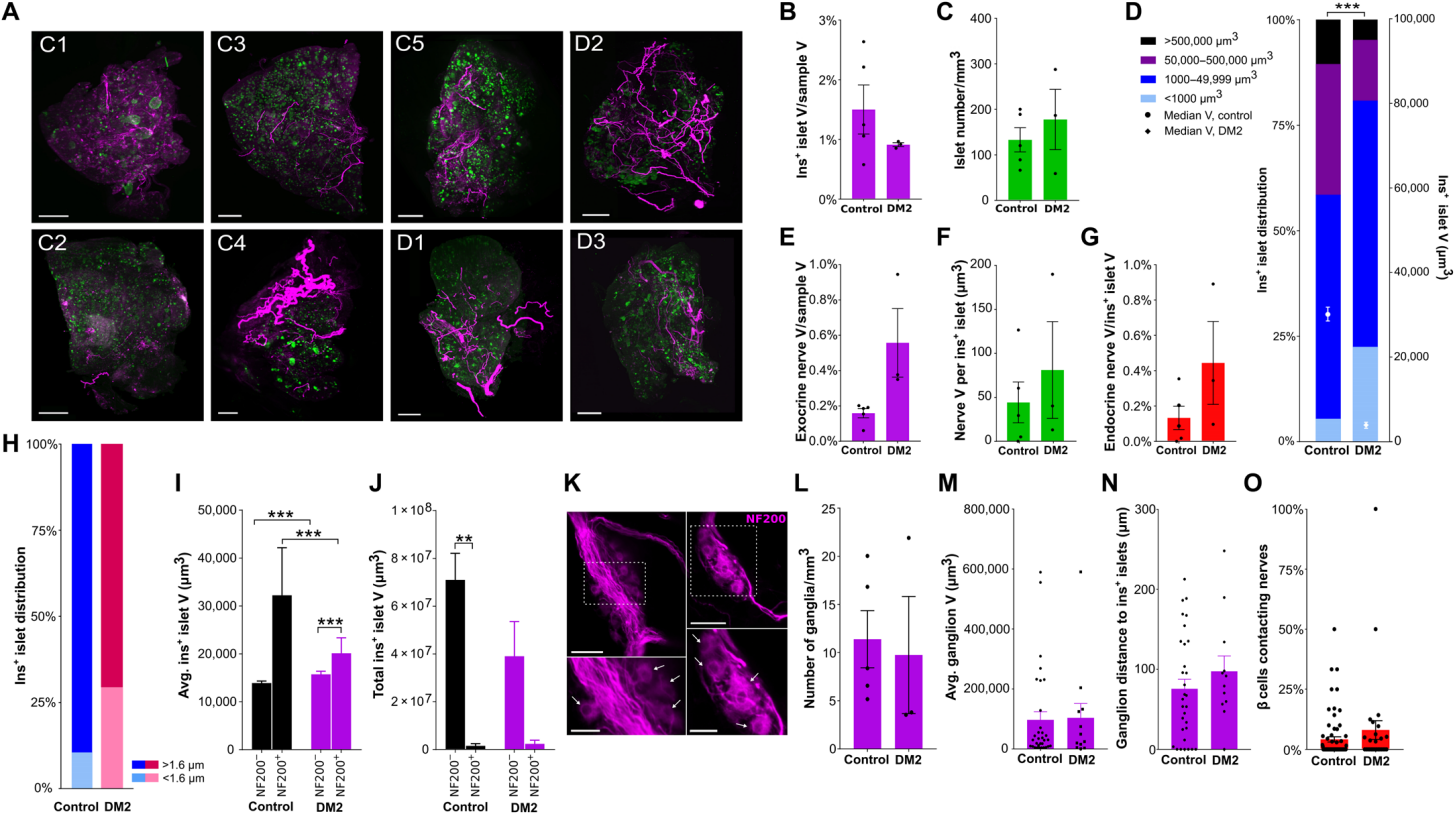
β cell distribution and innervation in human pancreatic samples. (**A**) Maximum projections of pancreatic samples from human donors without (C1 to C5) and with type 2 diabetes (DM2; D1 to D3) at 1.3×. Scale bars, 1000 μm. (**B**) β cell volume. (**C**) Insulin^+^ islets per cubic millimeter. (**D**) Insulin^+^ islet volume distribution (left axis) and median volume (right axis). (**E**) Exocrine nerve volume. (**F**) Endocrine nerve volume per insulin^+^ islet. (**G**) Endocrine nerve volume corrected for insulin^+^ islet volume. (**H**) Distribution of insulin^+^ islets located at <1.6 and >1.6 μm from nerves. Islets per group: 28,315 control and 6790 DM2. (**I**) Mean volume of insulin^+^ islets ± NF200^+^ innervation. Islets per group: 25,519/236/7448/345. (**J**) Total volume of insulin^+^ islets ± NF200^+^ innervation. (**K**) Intrapancreatic ganglia (NF200, magenta; confocal, 20×). Boxed areas magnified in lower panels with cell bodies indicated by arrows. Scale bars, 50 μm (top) and 25 μm (bottom). (**L**) Ganglia per cubic millimeter. (**M**) Volume of intrapancreatic ganglia. Ganglia per group: 31/12. (**N**) Distance between intrapancreatic ganglia and insulin^+^ islets. Ganglia per group: 31/12. (**O**) β cells contacting nerves per islet. Islets per group: 73/28. Data are shown as means ± SEM or as median ± 95% confidence interval where indicated. Analyses by unpaired *t* test (B to G and L to M), Mann-Whitney test (H), or Kruskal-Wallis with Dunn’s test (I to J). *P < 0.05, **P < 0.01, and ***P < 0.001. T, total; D, duodenal; S, splenic. *N* = 5 control and *N* = 3 DM2.

Islet innervation differs between species (*3*, *39*) and the 3D relationships between islets and pancreatic nerves in healthy versus diabetic patients remain largely unknown. To assess these, islets and NF200^+^ innervation were examined in small, cleared pancreatic samples from healthy human donors and donors with type 2 diabetes (T2D; Table 1) by light-sheet imaging to assess islet distribution and relationship to innervation (Fig. 8A and movies S18 and S19).

**Table 1:** Human donor information. CVA, cardiovascular accident; HbA1c, hemoglobin A1C; N/A, not applicable; PFA, paraformaldehyde; M, male; F, female.

**Islet** **preparation**	**1**	**2**	**3**	**4**	**5**	**6**	**7**	**8**
Unique identifier	HP-18157-01	HP-18294-01	HP-18310-01	HP-18330-01	HP-19333-01	HP-19078-01T2D	HP-19051-01T2D	HP-19317-01T2D
Donor age (years)	43	49	38	53	38	66	53	52
Donor sex (M/F)	F	M	M	F	M	F	M	M
Donor BMI (kg/m^2^)	34	30	28	26.3	25.3	30.3	30	24.8
Donor HbA1c	4.6	5.5	5.9	5.4	5.3	6.5	7.8	7.6
Origin/source of islets (a)	PRODO	PRODO	PRODO	PRODO	PRODO	PRODO	PRODO	PRODO
Islet isolation center	PRODO, Aliso Viejo, CA	PRODO, Aliso Viejo, CA	PRODO, Aliso Viejo, CA	PRODO, Aliso Viejo, CA	PRODO, Aliso Viejo, CA	PRODO, Aliso Viejo, CA	PRODO, Aliso Viejo, CA	PRODO, Aliso Viejo, CA
Donor history of diabetes? Yes/No	No	No	No	No	No	Yes	Yes	Yes
Diabetes duration (years)	N/A	N/A	N/A	N/A	N/A	Unknown	Unknown	6
Glucose- lowering therapy at time of death (b)	N/A	N/A	N/A	N/A	N/A	Diet	Oral medicine: Glipizide and Januvia	Metformin
Donor cause of death	Anoxia	Anoxia	Head trauma	Stroke	Head trauma	Anoxia	Cerebrovascular/ stroke	CVA
Warm ischemia time (hours)	Unknown	Unknown	Unknown	Unknown	Unknown	Unknown	Unknown	Unknown
Cold ischemia time (hours)	Unknown	Unknown	Unknown	Unknown	Unknown	Unknown	Unknown	Unknown
Estimated purity (%)	N/A	N/A	N/A	N/A	N/A	N/A	N/A	N/A
Estimated viability (%)	N/A	N/A	N/A	N/A	N/A	N/A	N/A	N/A
Additional notes	Postfixed in PFA	Postfixed in PFA	Postfixed in PFA	Postfixed in PFA	Postfixed in PFA	Postfixed in PFA	Postfixed in PFA	Postfixed in PFA

As expected, total β cell volume (Fig. 8B) and islet number (Fig. 8C) were highly variable (*40*). The β cell volume (as a percentage of the total pancreatic sample volume) was lower in the diabetic donors, varying between 0.47 and 2.2% in the control group and 0.85 and 0.97% in the diabetic group. Islet numbers ranged from 66 to 200 islets/mm^3^ in the control group to 58 to 287 islets/mm^3^ in the diabetic group. While islet number per cubic millimeter was greater in humans than in mice, the β cell volume (%) was very similar in murine and human tissues. The β cell volume distribution in nondiabetic pancreata was not significantly different to that in mice (Fig. 8D). Larger islets were disproportionately reduced, and the mean volume of an individual islet was significantly lower in diabetic compared to healthy individuals.

In human samples from healthy donors, NF200^+^ innervation was similar between endocrine (0 to 0.89% nerve volume per islet) and exocrine tissue (0.06% to 0.94% nerve volume per exocrine tissue; Fig. 8, E to G). Exocrine nerve volume, endocrine nerve volume per islet, islet nerve density, and proportion of innervated islets were greater in diabetic individuals (Fig. 8H). In nondiabetic individuals, innervated insulin^+^ islets are significantly larger than those without innervation, in line with the findings in mice, but innervated insulin^+^ islets are a smaller proportion of the total islet volume than seen in mouse pancreata. In diabetic individuals, innervated islets are significantly smaller than in nondiabetic individuals (Fig. 8, I and J).

We next assessed intrapancreatic ganglia in human pancreatic samples. Human intrapancreatic ganglia were larger than those found in mice (Fig. 8K), but ganglion density and distance from islets were similar to C57BL/6 and nondiabetic NOD mice. There was no significant difference in ganglia size between nondiabetic and diabetic donors (Fig. 8, L to N). Last, we examined contacts between NF200^+^ nerves and β cells. The proportion of β cells in contact with NF200^+^ fibers was half of that in control mice (4.15 versus 9.44%) and was preserved in individuals with diabetes (6.07%; Fig. 8O).

Together, β cell volume and distribution in human pancreata were comparable to murine pancreata, and innervated islets were significantly larger than noninnervated islets. In samples from individuals with diabetes, exocrine and endocrine innervation as well as proportion of innervated islets were increased, and nerve contacts with β cells persist.

### 3D analysis of sympathetic and parasympathetic innervation in optically cleared mouse pancreas

**Fig. 9:**
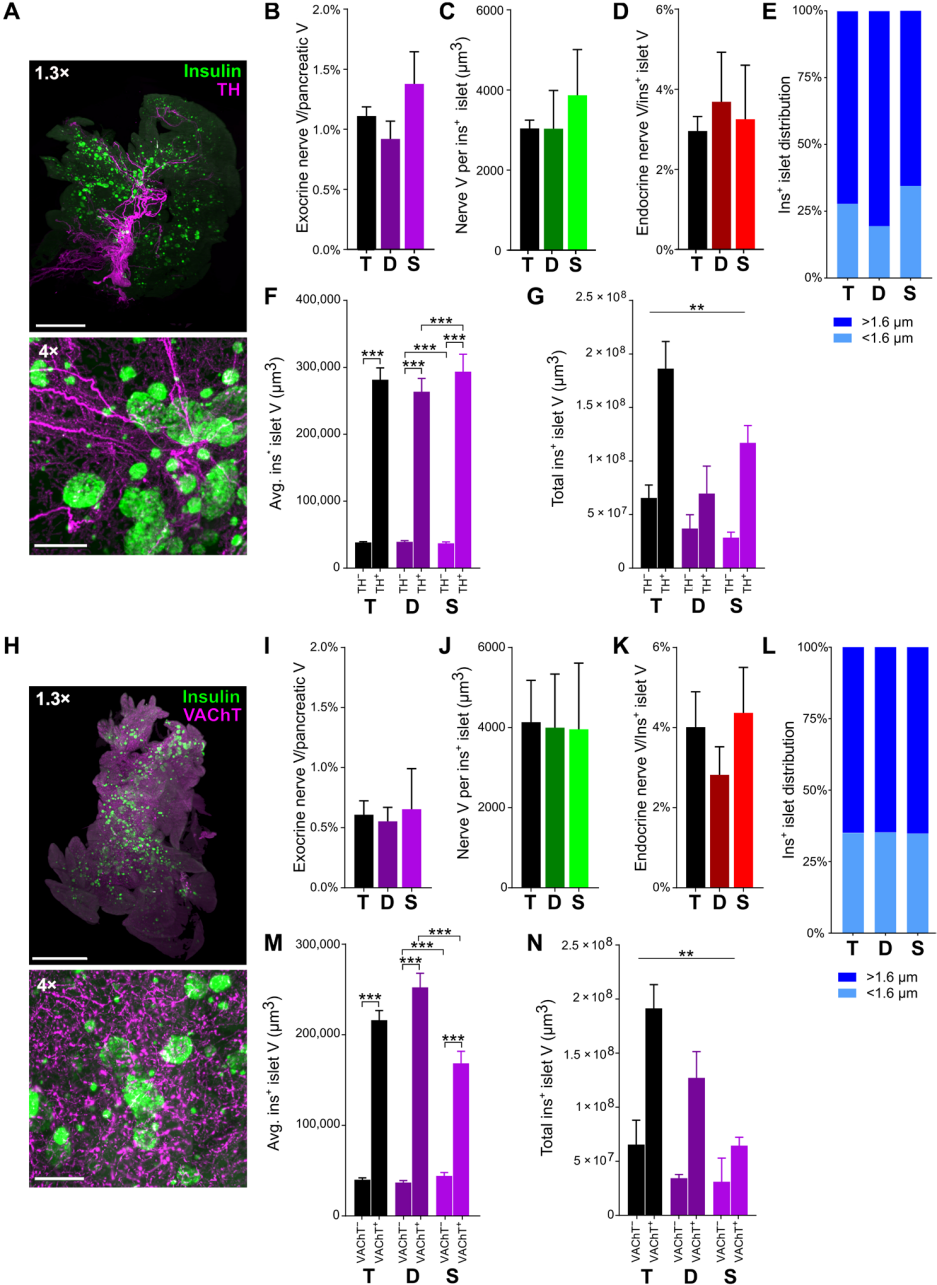
β cell distribution and sympathetic and parasympathetic innervation in C57BL/6 mice. (**A**) Maximum projections of TH and insulin. Scale bars, 2000 μm at 1.3× and 200 μm at 4×. (**B**) TH^+^ exocrine nerve volume. (**C**) TH^+^ endocrine nerve volume per insulin^+^ islet. (**D**) TH^+^ endocrine nerve volume by insulin^+^ islet volume. (**E**) Distribution of insulin^+^ islets located at <1.6 and >1.6 μm from TH^+^ nerves. Islets per group: 10,610/4773/5837. (**F**) Mean insulin^+^ islet volume for insulin^+^ islets with and without TH^+^ innervation. Islets per group: 8542/3314/4699/1320/3843/1994. (**G**) Total volume for insulin^+^ islets with and without TH^+^ innervation. (**H**) Maximum projections of VAChT and insulin. Scale bars, 2000 μm at 1.3× and 200 μm at 4×. (**I**) VAChT^+^ exocrine nerve volume. (**J**) VAChT^+^ endocrine nerve volume per insulin^+^ islet. (**K**) VAChT^+^ endocrine nerve volume corrected for insulin^+^ islet volume. (**L**) Distribution of insulin^+^ islets located at <1.6 and >1.6 μm from VAChT^+^ nerves. Islets per group: 12,661/7165/5496. (**M**) Mean volume for insulin^+^ islets with and without VAChT^+^ innervation. Islets per group: 8542/3314/4699/1320/3843/1994. (**N**) Total volume for insulin^+^ islets with and without VAChT^+^ innervation. Data are shown as means ± SEM or as median ± 95% confidence interval where indicated. Analyses by unpaired *t* test (B to E and I to L) or Kruskal-Wallis with Dunn’s test (F, G, M, and N). **P < 0.01 and ***P < 0.001. T, total; D, duodenal; S, splenic. *N* = 5.

The pancreas is composed of multiple cell types, is richly vascularized, and is densely innervated by sympathetic, parasympathetic, and sensory nerves. We wanted to compare our analyses of pancreatic innervation using the pan-neuronal marker, NF200, with pathway-specific pancreatic innervation. Using a modified iDISCO^+^ protocol specifically optimized for pancreatic tissue, we examined tyrosine hydroxylase (TH) immunolabeling to mark sympathetic nerve fibers (movie S20) and vesicular acetylcholine transporter (VAChT) immunolabeling to identify parasympathetic nerve fibers (movie S21) across the mouse pancreas. There was more TH^+^ (Fig. 9, A to D) and VAChT^+^ (Fig. 9, H to K) innervation than NF200^+^ innervation in both the exocrine and endocrine pancreas. In keeping with our findings examining NF200^+^ fibers, TH and VAChT nerve density were threefold to more than sixfold greater in the endocrine than in the exocrine pancreas. The proportion of islets containing or in contact with TH^+^ fibers was 27.7% (Fig. 9E) compared to 35.0% for VAChT^+^ fibers (Fig. 9L). In line with our findings with NF200, both TH^−^ (Fig. 9F) and VAChT^−^ (Fig. 7M) innervated islets were significantly larger than noninnervated islets, and as a result, the majority of insulin^+^ islet volume is composed of innervated islets (Fig. 9, G and N).

The optimized iDISCO^+^ protocol was also effective for labeling fibers expressing TRPV1 (transient receptor potential cation channel, subfamily V, member 1) and synapsin (fig. S4). In addition, we applied a novel alternative approach to visualize islet vasculature by combining insulin immunolabeling with fluorophore-tagged dextran or CD31 antibody, followed by optical clearing using ethyl cinnamate (ECi) to preserve fluorescence while allowing for additional immunostaining tissue (fig. S4) (*41*). These data demonstrate that modification of optical clearing protocols allows for visualization of multiple markers in pancreatic tissue.

In summary, NF200, TH, and VAChT immunostaining all demonstrate that large islets have enriched islet innervation and that large innervated islets represent at least half of total pancreatic islet volume. However, analysis of pancreatic innervation using TH and VAChT immunostaining suggests that endocrine nerve density is greater than revealed by NF200 immunostaining.

## DISCUSSION

Tissue clearing, 3D imaging, and unbiased image analysis have been widely used in the CNS to provide new insights into anatomical pathways and patterns of regional activation. However, there have been few applications in peripheral organs such as the pancreas. Whole-organ clearing and imaging are especially suited for the mapping of filamentous structures, particularly to delineating innervation across large distances that can be difficult to achieve using traditional serial sections and 2D imaging. Tissue clearing has been used previously in thick pancreatic sections (350 to 1000 μm) (*9*, *10*, *12*, *42*–*44*) and small pieces of pancreatic tissue (*11*) and then imaged with optometry or high-magnification confocal microscopy for detailed analysis [see (*45*–*47*) for review]. This has provided important information about islet characteristics and structural relationships over a close range. In particular, previous studies using 3D imaging in pancreatic sections, fetal tissue, and young mice have provided data about islet innervation (*9*, *10*, *13*, *14*, *42*, *48*). Our data extend these important published studies. Different pancreatic regions have diverse embryological origins and variations in islet density and function and are supplied by neurons from different extrapancreatic ganglia. Without assessing pancreatic structure across the whole organ, our understanding and quantification of pancreatic anatomy, including possible regional differences, are incomplete and possibly inaccurate.

Tissue clearing and volume imaging of the pancreas provided several new insights. Innervation of the endocrine pancreas is significantly enriched compared to the surrounding exocrine pancreas, with marked regional variation. Islets are closely associated with pancreatic innervation, and innervated islets are significantly larger than noninnervated islets, in both mouse and human. Intrapancreatic ganglia are sparse and close to islets. Almost half of α cells and a tenth of β cells contact NF200^+^ fibers, irrespective of islet size or location. Last, islet nerve density and expression of NF200 are increased in the remaining islets of two mouse models of T1D, with temporal and regional differences, and greater in human T2D, in keeping with nerve remodeling.

3D imaging across the whole pancreas provides straightforward measurement of multiple islet characteristics and identifies significant regional differences that would be laborious or impossible to obtain by serial sectioning. We readily measured β cell volume across multiple pancreata, and our findings are in agreement with previous studies at 1 to 2% in mouse pancreas (*24*) and at 1 to 4% in human pancreas (*49*, *50*). Increased islet volume in the splenic pancreas is in keeping with previous observations using 2D histology, in isolated islets and in transgenic mice (*51*, *52*). Intensity of insulin immunostaining was significantly lower in the splenic pancreas. The splenic pancreas contains significantly larger islets, and previous studies report lower insulin immunostaining intensity and fewer insulin granules in large islets, while other studies demonstrate lower c-peptide content in β cells from the splenic pancreas (*53*, *54*). Our approach also facilitates rapid analysis of islet volume distribution across the pancreas. The majority of islets were between 1000 and 500,000 μm^3^, equivalent to islet diameters of 12 to 98 μm (assuming spherical islets), with around a fifth of islets having volumes larger than 500,000 μm^3^. Whole-organ imaging demonstrated significant differences in islet biology between diabetes models. Islet number and β cell volume were reduced in diabetic NOD mice, with the intensity of insulin immunostaining relatively preserved in some islets and a notable shift to small islets. Islet number and volume were also reduced with STZ treatment, but intensity of insulin immunostaining was markedly reduced, and size distribution was minimally altered. Together, these observations validate tissue clearing and 3D imaging as a reliable straightforward method to assess β cell volume and other characteristics across the entire pancreas.

Whole-tissue 3D imaging confirmed dense pancreatic innervation and revealed markedly greater nerve density in the endocrine pancreas, over sixfold greater than in the exocrine pancreas of mice. These findings confirm the results of previous studies reporting close association between islets and nerves using 2D histology and 3D examination of pancreatic sections (*3*, *9*, *10*). We extended these findings to show that endocrine NF200^+^ innervation was not uniform throughout the pancreas but enriched in the duodenal portion. These regional differences may reflect the distinct embryological origins of the duodenal and splenic regions. Further studies will determine whether regional differences in pancreatic innervation contribute to reported regional differences in islet composition, size, function, and susceptibility to immune loss.

3D analysis of islets and innervation across the whole pancreas revealed previously unknown features of the close anatomical relationship between islets and nerves. In mice and human samples, innervated islets are a relatively small fraction of all islets by number, but they are, on average, 10-fold larger than noninnervated islets. As a result, innervated islets represent around half of the total β cell volume. The large volume of innervated islets is in accordance with a role for neural signals in islet development and maintenance. In both mice and zebrafish, β cells aggregate close to pancreatic nerves in development, and islet architecture is disrupted by loss of neural signals (*55*, *56*). There is also close physical association between nerves and islets in human embryos, particularly in the middle and late trimester, when there is rapid development of the endocrine pancreas (*55*). Less is known about the role of neural signals in β cell maintenance, but vagotomy reduces β cell replication in rats (*57*). Vagotomy disrupts the afferent and efferent signals to several intra-abdominal organs, so further studies are needed to determine whether loss of neural signals, specifically to the pancreas, disrupts islet structure and function during development and after birth.

Islets are closely associated with the pancreatic duct and highly vascularized, so it is possible that the proximity between nerves and islets is related to innervation of the duct or vessels. Islet blood vessels are richly innervated, and neural signals have marked effects on islet blood flow (*58*). However, several studies suggest that vascular signals actually reduce endocrine cell differentiation during development, and in zebrafish, islets remain densely innervated even in the absence of islet vascularization.

Although NF200-innervated islets are large, on average, NF200 immunostaining intensity is greater in smaller innervated islets. NF200 expression has been linked to nerve diameter and conduction velocity, so it is possible that differences in NF200 immunostaining intensity may have functional consequences (*29*, *30*). In human pancreata, small islets have a greater proportion of β cells compared to other endocrine cell types and higher insulin content (*59*). Similarly, small rat islets were functionally superior to larger islets in ex vivo studies and after transplantation (*60*). In keeping with previous work (*3*), NF200^+^ nerves contact a small proportion of β cells in each islet, with no proportional differences between pancreatic regions or islet sizes. The proportion of innervated β cells is similar to the percentage of β cells that are reported to act as hub cells in the islets, and hub cells are reported to be modulated by cholinergic agonists (*61*). Although a minority of β cells contact NF200^+^ nerves, neural signals could influence activity across multiple β cells through electrical coupling. In contrast, and in keeping with previous work (*3*), NF200^+^ nerves contact a much greater proportion of α cells, which lack gap junctions. Whether variation in NF200 immunostaining intensity or proximity of individual β cells to nerves contributes to functional heterogeneity of β cells is currently unknown and warrants further studies.

The development of diabetes in NOD mice and in STZ-treated mice is associated with rapid and significant increases in islet nerve density. In NOD mice, increased nerve/islet volume suggests that nerve volume may be preserved, while β cell volume is reduced in surviving islets. The proportion of innervated α cell clusters increased in diabetic NOD mice. In STZ-treated mice, nerve volume per islet, nerve density per islet, and nerve density in α cell clusters are all increased. These findings suggest that increased nerve density is restricted to the remaining insulin^+^ islets in NOD mice, while both α cell and β cell nerve density is increased in STZ-treated mice. The increases in insulin^+^ islet nerve density in diabetic NOD and STZ-treated mice are similar in magnitude to nerve density changes in response to physiologically relevant stimuli. In the CNS, fasting leads to an almost twofold increase in agouti related neuropeptide (AgRP)/neuropeptide Y–positive (NPY^+^) terminals in the paraventricular nucleus, a major pathway regulating food intake (*62*). In the peripheral nervous system, skin inflammation increased sensory nerve density twofold and was associated with increased sensitivity to thermal and mechanical stimuli (*63*). Further studies will be required to test the functional consequences of increased islet nerve density.

The intensity of NF200 immunostaining was significantly increased in the islets of diabetic NOD and STZ-treated mice. NF200 staining intensity increases in response to nerve regeneration (*20*, *21*), so up-regulation of NF200 may reflect ongoing regeneration of islet innervation. These findings, and the time course of the increased nerve density with STZ treatment, are highly suggestive of nerve regeneration; reported rates of nerve regrowth after crush injury are up to 4 mm/day, and restoration of electrical activity in peripheral nerves after chemical injury occurs within days. Our findings may be responses to STZ directly, to hyperglycemia, and to inflammatory processes and/or interactions between endocrine cells and neurons to regulate neural density. Increased nerve density is reported in response to inflammation in several tissues and their adjacent structures, and these changes can either be protective or exacerbate inflammation (*64*, *65*). Increased insulin^+^ islet nerve density in NOD mice, and the increase in exocrine and endocrine nerve density in STZ-treated mice, may reflect the major sites of immune activity/inflammation. Both hyperglycemia and STZ treatment increase β cell production of nerve growth factor (NGF) (*66*). Its receptor, tyrosine kinase receptor A (TrkA), is expressed on both sympathetic and sensory nerves, and previous 2D imaging studies report that sensory and sympathetic nerve density are increased with STZ treatment (*33*). In previous studies, NGF overexpression in β cells significantly increased sympathetic islet innervation (*67*). The cross-talk between nerves and islets in healthy versus diabetic tissue remains largely unstudied.

There is considerable variability in the reported changes in α cell mass in models of diabetes. In our studies in diabetic NOD mice, α cell volume was not significantly higher as a proportion of total pancreatic volume, but the ratio of β to α cell volumes was significantly increased (Fig. 4, B and C). Similar to our findings, Plesner *et al.* (*68*) describe a non-significant increase in α cell mass in prediabetic and diabetic NOD mice and a significant increase in the proportion of α cells/islet area in diabetic NOD mice. Our longitudinal studies show changes in α cell volume with time after STZ treatment, with a significant decrease 2 weeks after the completion of treatment. This is in keeping with previous studies (*69*), but the α cell response to STZ treatment has also been described to increase (*70*), to remain unchanged (*71*), or to vary with time after STZ treatment (*72*).

In our studies, α and β cell contacts are maintained in diabetic NOD and STZ-treated mice. One limitation of our assessment is that we quantify the number of endocrine cells contacting nerves, but we cannot quantify the number of contacts per endocrine cell. It is possible that the number of contacts with β or α cells is modified in diabetes. Sympathetic fibers also contact delta cells and vasculature to a lesser extent (*3*) in mouse islets. Further work is needed to determine whether NF200^+^ fibers contact delta cells or other islet structures and the effects of diabetes and STZ treatment on these contacts.

Our results examining innervation in NOD mice are consistent with recent 3D imaging of thick pancreatic sections (*9*, *13*) reporting regions with increased innervation in these mice. Prior 2D studies have reported loss of islet sympathetic innervation in NOD mice (*35*), but these studies used different neural markers and examined NOD mice with longer duration of diabetes. It is possible that the increased islet innervation in NOD mice we observe is lost with increasing duration of diabetes. Alternatively, there may be pathway-specific changes such that sympathetic innervation is reduced, but parasympathetic and/or sensory innervation are increased, leading to an increase in NF200^+^ innervation found in our studies. Future studies using iDISCO^+^ will be required to dissect the longitudinal changes and contribution of specific neural pathways in mouse models of T1D, as well as the associations between innervation and immune infiltration.

There are several differences between mouse and human islets as well as similarities between these species. Human β cell volume (%) was similar to the proportion of insulin^+^ islets in C57BL/6 mice. Islet size distribution was also remarkably similar between mouse and human pancreata. Similar to the findings in mice, intrapancreatic ganglia in human tissue are sparse and close to islets, but they are markedly larger, of the order of 200 neurons on average. There have been conflicting results about islet innervation in human versus murine samples. In our studies, the proportion of innervated islets and the finding that innervated islets were larger than noninnervated were similar in both humans and mice. Initial 2D imaging reported reduced islet innervation in human samples, but recent data from optically cleared human samples using markers for sympathetic nerves suggest that human islets, like mouse islets, have a dense neural network (*9*, *11*). In keeping with previous studies examining TH^+^ fibers in human pancreatic samples (*3*, *11*), innervation density is similar in human exocrine and endocrine pancreas. We found that NF200^+^ innervation is present in human islets, in keeping with previous studies demonstrating TH^+^ fibers in islets (*3*, *11*), but at a lower density than mouse islets. A smaller proportion of human β cells contact NF200^+^ fibers compared to mouse islets.

There are also similarities between innervation in STZ-treated mice and in samples from T2D individuals. In pancreata from T2D donors, nerve volume per islet, nerve density, and the proportion of innervated islets are all increased. The human tissue samples we analyzed provide a snapshot of islets and innervation from postfixed tissue as well as from individuals with variable comorbidities, age, and time from death. These factors likely contribute to the sample variability, in line with previous human data (*73*). In aggregate, our data suggest that islet innervation is present in human islets, albeit at lower levels than mouse islets, and innervation appears to be at least preserved, possibly increased, in human T2D individuals. The increase in exocrine innervation in pancreatic tissue from T2D donors may reflect more generalized pancreatic pathology that is increasingly recognized as a feature of T2D. One limitation of our studies is that we did not examine insulin^−^ islets by glucagon staining in the human samples, so it is unknown whether whole islet nerve density or α cell contacts are altered in T2D. Further studies examining specific neural pathways and further endocrine cell types in human pancreatic tissue are required to fully assess normo- and pathophysiological species differences.

Our studies using NF200 as a neural marker do not differentiate between parasympathetic, sympathetic, and sensory fibers. Using an optimized iDISCO^+^ protocol, we examined sympathetic and parasympathetic innervation in wild-type mice, and many findings mirror those seen with NF200^+^ innervation. Similar to our findings using NF200, both sympathetic and parasympathetic endocrine innervation are enriched compared to exocrine innervation, and innervated islets are significantly larger than noninnervated islets. These findings differ from published studies that show similar TH^+^ innervation density in exocrine and endocrine tissue (*74*). However, previous reports analyzed innervation in female mice sampling cryosections every 400 μm rather than innervation across the whole pancreas. The proportion of innervated-to-noninnervated islets is also greater when assessed using sympathetic and parasympathetic markers. The distribution of TH^+^ and VAChT^+^ innervation differs, with several large volume TH^+^ fibers contributing to higher TH^+^ volume compared to VAChT^+^ innervation in the exocrine pancreas. In keeping with previous reports in adult mice (*75*, *76*), we observed occasional TH^+^ β cells. We excluded these, as far as possible, based on their morphology, volume, and overlap with insulin immunostaining, but it is conceivable that our estimate of TH^+^ innervation may be an overestimate. TH^+^ and VAChT^+^ endocrine innervation are higher than for NF200. However, while NF200 has been reported to be expressed in a wide range of myelinated and unmyelinated fibers, our results and previous studies suggest that NF200 does not label all fibers (*19*), and it is possible that we may have overlooked alterations in NF200^−^ fibers in our studies. Alternative pan-neuronal markers have significant limitations. For example, protein gene product 9.5 (PGP9.5) is expressed in islet endocrine cells and innervation. Pathway-specific markers are also imperfect. TH labels most, but not all, sympathetic nerve fibers since there are also populations that are TH^−^ but express NPY (*77*). VAChT immunostaining is primarily in the terminal neuronal arborization and so visualizing larger cholinergic nerve fibers may be incomplete (*78*). While there are similarities among innervation patterns with NF200, TH, and VAChT, our studies do not allow us to determine whether the changes in pancreatic innervation with diabetes are generalized or specific to sympathetic, parasympathetic, or sensory pathways. Future work will assess important pathway-specific changes in pancreatic innervation and their contacts with specific endocrine cell types in both mouse and human metabolic disease. One disadvantage of iDISCO^+^ is that it does not preserve endogenous fluorescence. Therefore, we also developed and validated a novel alternative approach that combines immunostaining and tissue clearing with ECi for use in adult murine tissues (*41*). This preserves endogenous fluorescent signals while allowing for antibody labeling of additional targets. ECi clearing also provides a less toxic alternative to iDISCO^+^ (*41*). A combination of fluorescently tagged dextran to delineate blood vessels, immunostaining for innervation and islets, and ECi tissue clearing will allow us to further assess the organ-wide association between innervation, islets, and vasculature.

In summary, we have used whole-organ tissue clearing and imaging to create a 3D atlas mapping islets and innervation across the pancreas as a tool to quantify β cell mass, define islet characteristics, map pancreatic innervation, and assess the anatomical interaction between islets and innervation in healthy and diabetic mice and humans. This approach demonstrates dense islet innervation and identifies distinguishing features of innervated islets and the regional differences. Such regional variations illustrate the importance of whole-organ imaging when assessing pancreatic anatomy. Our studies confirm that innervation is present in human islets and directly contacts β cells. We demonstrate that islet innervation is markedly increased in diabetic NOD mice, STZ-treated mice, and likely in diabetic human pancreata. In combination with up-regulation of NF200 immunostaining, this suggests increased rapid reorganization of pancreatic innervation and possible nerve growth within islets. Future studies will identify the neurochemical characteristics, time course, and functional consequences of these changes. Intrapancreatic ganglia and nerve contacts in islets are maintained in diabetes. The tissue clearing and imaging approaches we have used and optimized are broadly applicable to investigating pancreatic structures and innervation in other diseases, such as pancreatitis and pancreatic cancer, and are relevant to imaging vasculature and innervation in other organs. Our data also have important translational implications. Our data suggest that the close association between islets and pancreatic nerves is maintained in human T2D; therefore, the anatomical pathways that would allow for targeted neuromodulation to regulate pancreatic function are preserved. Defining pancreatic neurocircuitry is crucial to understanding neural regulation of pancreatic function, as it elucidates anatomical pathways for direct effects on endocrine cells. Future studies will determine critical interactions between β cells and nerves, whether variation in islet innervation density is associated with differences in islet function, and whether metabolic disease leads to functional deficits in islet innervation independent of structure.

## MATERIALS AND METHODS

### Animals

Ad libitum fed C57BL/6 mice were maintained under controlled conditions (12-hour light/12-hour dark cycle, 22°C). NOD mice (NOD/ShiLtJ, the Jackson Laboratory, Bar Harbor, ME, USA) and STZ-treated mice were used to model T1D. Female NOD mice aged 12 to 16 weeks with two consecutive blood glucose measurements of >300 mg/dl (morning, nonfasting) were termed diabetic. Littermates with blood glucose <200 mg/dl were used as nondiabetic controls. Multiple low-dose STZ-treated mice (males, aged 10 weeks) were generated by treating C57BL/6N mice (Charles River, Wilmington, MA, USA) intraperitoneally with freshly made STZ (40 mg/kg; Sigma-Aldrich, St. Louis, MO) in citrate-saline buffer (pH 4.5) for five consecutive days and euthanizing them at 5 or 15 days following the final STZ injection. Non–STZ-treated littermates were used as controls. All protocols were approved by the Institutional Animal Care and Use Committee.

### Sample collection

Mice were anesthetized with isoflurane (3%) and perfused with heparinized saline followed by 4% paraformaldehyde (PFA; Electron Microscopy Sciences, Hatfield, PA, USA). Pancreata were dissected, cleared of adipose tissue, divided into duodenal and splenic regions (Fig. 1A), with the gastric lobe included with the duodenal lobe, and postfixed overnight in 4% PFA at 4°C. For antibody evaluation experiments, small pancreatic samples (2 to 3 mm diameter) were assessed. On the following day, the tissue was washed in phosphate-buffered saline (PBS; 3×) before proceeding with optical clearing protocols.

Human samples (Table 1) were obtained from Prodo Laboratories Inc. (Aliso Viejo, CA, USA) and postfixed in 4% PFA. Since human samples were processed upon acquisition and not simultaneously as with mouse tissue, we could not compare staining intensity between samples. All samples were harvested from the superior margin of the tail of the pancreas.

### Optical clearing of mouse and human pancreata

Whole-organ staining and clearing were performed using iDISCO^+^ (*15*). Dissected pancreata were dehydrated [20, 40, 60, 80, and 100% methanol at room temperature (RT)], delipidated [100% dichloromethane (DCM; Sigma-Aldrich, St. Louis, MO, USA)], and bleached in 5% H_2_O_2_ (overnight, 4°C). Pancreata were rehydrated (80, 60, 40, and 20% methanol) and permeabilized [5% dimethyl sulfoxide/0.3 M glycine/0.1% Triton X-100/0.05% Tween-20/0.0002% heparin/0.02% NaN_3_ in PBS (PTxwH)] for 1 day. Pancreata were then placed in blocking buffer [PTxwH + 3% normal donkey serum (Jackson ImmunoResearch, West Grove, PA, USA)] at 37°C overnight. Samples were incubated with primary antibodies (table S1) in blocking buffer for 3 or 6 days (small pancreatic pieces and hemipancreata, respectively) at 37°C. After five washes with PTxwH at RT (final wash overnight), samples were incubated with secondary antibodies in blocking buffer (1:500) for 3 or 6 days. Samples were washed with PTxwH (five times, RT) and PBS (five times, RT), dehydrated with a methanol gradient, then washed in 100% methanol (three times, 30 min each) and DCM (three times, 30 min each), and then transferred to dibenzyl ether (DBE; Sigma-Aldrich) to clear. Primary antibody specificity was confirmed in pancreatic tissue from reporter mice expressing tdTomato in defined neural populations. There was no immunolabeling without primary antibodies using iDISCO^+^ or ECi. A modified iDISCO^+^ protocol used 0.5% Triton X-100 and 0.1% Tween-20 for the permeabilization, blocking, and primary and secondary antibody buffers.

A modified ECi tissue clearing protocol was used for samples from animals injected intravenously with fluorophore-tagged dextran (100 μm, 25 mg/ml) or a direct conjugated CD31 antibody (100 μm, 50 mg/ml). Tissue was harvested, postfixed, and washed with PBS as described above. Samples were incubated with 3% H_2_O_2_ (10 min, RT), washed in PBS with 0.2% Triton X-100 (Ptx2; three times over 3 h, RT), and incubated overnight in PTx2 + heparin (10 mg/ml; PTwH) and 3% normal donkey serum at RT. Samples were incubated with primary antibodies in PTwH with 3% normal donkey serum (2 days, RT) followed by PTwH washes (four times over 4 hours). Samples were incubated with secondary antibodies in PTwH with 3% normal donkey serum (2 days, RT), followed by PTwH washes as above. Optical clearing was achieved by incubating samples in 50% ethanol, 70% ethanol, 100% ethanol (all pH 9, 4 hours, 4°C), 100% ethanol (pH 9, overnight, 4°C), and finally one wash and one overnight incubation (RT) in ECi (Sigma-Aldrich) before imaging.

### Imaging processing and analysis

Z-stacked optical sections were acquired with an UltraMicroscope II (LaVision BioTec, Bielefeld, Germany; ×1.3, ×4, or ×12 magnification with dynamic focus with a maximum projection filter). Human samples were imaged at 1.3× with dynamic focus and with multiple Z-stacks acquired at 4× with 20% overlap and tiled using the plugin TeraStitcher through the ImSpector Pro software (LaVision BioTec). Spatial resolutions of light-sheet images were 5 μm by 5 μm by 5 μm at 1.3×, 1.63 μm by 1.63 μm by 5 μm at 4×, and 0.602 μm by 0.602 μm by 2 μm at 12×.

Small mouse pancreatic sections were imaged in glass-bottom eight-well chambers (Ibidi, Gräfelfing, Germany) filled with immersion media DBE or ECi and imaged using an inverted Zeiss LSM 880 confocal microscope with a 10× [numerical aperture (NA), 0.3] objective and a step size of 5 μm. Spatial resolution for confocal images acquired at 10× was 1.67 μm by 1.67 μm by 5 μm.

Imaris versions 9.1 to 9.3.1 (Bitplane AG, Zürich, Switzerland) were used to create digital surfaces covering the islets (1.3× and 4× images) and innervation (4× images) to automatically determine volumes and intensity data. Volume reconstructions were performed using the surface function with local contrast background subtraction. For detection of islets, the threshold factor corresponded to the largest islet diameter in each sample. For detection of nerves, the threshold factor was set to 12.2 μm. A smoothing factor of 10 μm was used for islets analyzed at 1.3, and a factor of 3.25 μm was used for analysis of islets and nerves at 4×. For detection of TH^+^ nerves, TH^+^ β cells (*75*, *76*) were manually removed from the final TH^+^ nerve surface by excluding volumes below 120 μm^3^ residing within insulin^+^ islets and overlapping with insulin staining. The Imaris Distance Transform Matlab XTension function was used to calculate the distance of each islet surface from the innervation surface. Distances of islets are reported as the intensity minimum of the distance transformation channel (intensity 0 = islet touching nerve) for each islet surface to the nerve surface as calculated by the distance transformation operation. In confocal images, digital surfaces were created to cover nerves and individual β cells, α cells, or ganglia. For detection of ganglia, a region of interest was manually created around each individual ganglion to create a digital surface specifically covering cell bodies, but not nerve fibers. The Imaris Distance Transform Matlab XTension was then used as above to determine the distance between ganglia and insulin^+^ islets or the distance between nerves and individual α or β cells with a distance of 0 indicating a nerve contact. Limitations to our analyses of endocrine cell contacts include the following: We may not have captured α/β cells with lower staining intensity; in some cases, we could not completely separate adjacent endocrine cells and therefore counted multiple adjacent cells as a single cell; our method quantifies the number of endocrine cells contacting nerves but does not allow for quantification of number of contacts per endocrine cell.

### Statistical analyses

Data are shown as means ± SEM. Distribution was assessed by Shapiro-Wilk test. Significance was determined by unpaired two-way *t* test or one-way analysis of variance (ANOVA) with post hoc Tukey’s multiple comparisons test (Gaussian distribution), Mann-Whitney test, or Kruskal-Wallis test followed by Dunn’s multiple comparisons test (nonparametric distribution). Significance was set at an α level of 0.05.

## Supplementary Material

aaz9124_Movie_S15.mp4

aaz9124_Movie_S14.mp4

aaz9124_Movie_S19.mp4

aaz9124_Movie_S11.mp4

aaz9124_Movie_S5.mp4

aaz9124_Movie_S21.mp4

abd3649_Movie_S3.mp4

abd3649_Movie_S1.mp4

abd3649_Movie_S5.mp4

aaz9124_Movie_S3.mp4

aaz9124_Movie_S2.mp4

aaz9124_Movie_S6.mp4

aaz9124_Movie_S1.mp4

aaz9124_Movie_S18.mp4

aaz9124_Movie_S20.mp4

aaz9124_Movie_S8.mp4

aaz9124_Movie_S12.mp4

aaz9124_SM.pdf

abd3649_Movie_S2.mp4

abd3649_Movie_S4.mp4

aaz9124_Movie_S4.mp4

aaz9124_Movie_S10.mp4

aaz9124_Movie_S7.mp4

aaz9124_Movie_S16.mp4

aaz9124_Movie_S17.mp4

aaz9124_Movie_S13.mp4

aaz9124_Movie_S9.mp4

## SUPPLEMENTARY MATERIALS

Supplementary material for this article is available at http://advances.sciencemag.org/cgi/content/full/6/41/eaaz9124/DC1

## Appendix

View/request a protocol for this paper from *Bio-protocol*.
