# Supplementary material for: A 3D atlas of the dynamic and regional variation of pancreatic innervation in diabetes
Source: Sci Adv. 2020 Oct 9;6(41):eaaz9124. doi: 10.1126/sciadv.aaz9124 (PMC7557000; doi:10.1126/sciadv.aaz9124)
Supplement: aaz9124_SM.pdf [file aaz9124_SM.pdf]

Science Advances
AAAS

advances.sciencemag.org/cgi/content/full/6/41/eaaz9124/DC1

Supplementary Materials for

A 3D atlas of the dynamic and regional variation of pancreatic innervation in diabetes

Alexandra Alvarsson, Maria Jimenez-Gonzalez, Rosemary Li, Carolina Rosselot, Nikolaos Tzavaras, Zhuhao Wu, Andrew F. Stewart, Adolfo Garcia-Ocaña, Sarah A. Stanley*

*Corresponding author. Email: sarah.stanley@mssm.edu

Published 9 October 2020, *Sci. Adv.* **6**, eaaz9124 (2020)
DOI: 10.1126/sciadv.aaz9124

The PDF file includes:

Figs. S1 to S4
Table S1

Other Supplementary Material for this manuscript includes the following:

(available at advances.sciencemag.org/cgi/content/full/6/41/eaaz9124/DC1)

Movies S1 to S21

Supplementary Figures

Figure S1. β-cell distribution and innervation in C57Bl/6 mice.

A) Distribution of insulin+ islets at increasing distance from the nearest nerve (left axis) and median distance from the nearest nerve (right axis) in C57BL/6 mice. P<0.0001, Mann-Whitney test. N=5, islets/group: 25310, 10030 and 15280.

B) Mean volume of insulin+ islets located <1.6 μm or >1.6 μm from the nearest nerve across the entire pancreas (T), in the duodenal (D) and splenic (S) regions. Kruskal-Wallis test: p<0.0001. Dunn’s test: Total <1.6 μm *vs*. >1.6 μm: p<0.0001; D <1.6 μm *vs*. >1.6 μm: p<0.0001; S <1.6 μm *vs*. >1.6 μm: p<0.0001; D >1.6 μm *vs.* S >1.6 μm: p<0.0001; N=5 samples, islets/group: 770, 11885, 325, 4690, 445, 7195.

C) Proportion of innervated (NF200+) insulin+ islets with volumes <10,000 μm^3, 10,000–49,999 μm^3, 50,000–500,000 μm^3 or >50,000 μm^3.

D) Number of β-cells contacting NF200+ nerves per insulin+ islet based on size. Small: <10 β-cells, medium: 10-100 β-cells, large >100 β-cells. Kruskal-Wallis test: p=0.0175. Dunn's test: p=0.0103, small *vs*. large.

E) Percentage of β-cells contacting NF200+ nerves per insulin+ islet based on size. Small: <10 β-cells, medium: 10-100 β-cells, large >100 β-cells. Kruskal-Wallis test: p=0.3041.

F) Large and thin nerve fibers expressing NF200 (magenta).

G) Analysis of innervation (yellow) and β-cells (red) (left panel) and the detection of nerve/β-cell contacts by distance transformation (left panel). β -cell clusters in cyan are touching the nerve surface (distance: 0).

Data are shown as mean±SEM, or as median±95% confidence interval where indicated. T: Total; D: Duodenal; S: Splenic.

Figure S2. β-cell distribution and innervation in nondiabetic and diabetic NOD mice.

A) Correlation between insulin+ islet number (left axis) and β-cell volume (right axis) and blood glucose in NOD mice. Islet number, T: p=0.0009; D: p=0.015; S: p=0.0034; Islet V, T: p=0.0021; D: p=0.0010; S: p=0.0137, linear regression. N=14.

B) Correlation between blood glucose and exocrine nerve volume. T: p=0.9520, D: p=0.7351, S: p=0.5316, linear regression. N=15.

C) Correlation between blood glucose and endocrine nerve volume corrected for islet volume (right axis) and endocrine nerve volume per insulin+ islet (left axis). Nerve V/Islet V: T: p=0.1540; D: p=0.2217; S: p=0.0436; Nerve V per islet, T: p=0.1784; D: p=0.4104; S: p=0.2396, linear regression. N=15.

D) Distribution of insulin+ islets at increasing distance from the nearest nerve (left axis) and median distance from the nearest nerve (right axis) in NOD mice. Kruskal-Wallis test: p<0.001; Dunn's test: nondiabetic S *vs*. diabetic S: p<0.001; nondiabetic D *vs*. S: p<0.001; diabetic D *vs*. S: p<0.001. N=8 nondiabetic, 7 diabetic. Islets/group: 9616, 5486, 4130, 4739, 3473, 1293.

E) Mean volume of insulin+ islets <1.6 µm and >1.6 µm from the nearest nerve in NOD mice. Kruskal-Wallis test: p<0.001, Dunn's test, <1.6 µm: nondiabetic T *vs*. diabetic T: p<0.001; nondiabetic D *vs*. nondiabetic S: p<0.001; nondiabetic S *vs*. diabetic S: p<0.001; >1.6 µm: nondiabetic T *vs*. diabetic T: p<0.001; nondiabetic D *vs*. S: p<0.001; nondiabetic D *vs*. diabetic D: p<0.001; diabetic D *vs*. S: p=0.0096. N=8 nondiabetic, 7 diabetic. Islets/group: 901, 4585, 500, 3605, 312, 3163, 199, 1187, 1401, 8190, 511, 4350.

F) Proportion of innervated (NF200+) insulin+ islets with volumes <10,000 μm^3, 10,000–49,999 μm^3, 50,000–500,000 μm^3 or >50,000 μm^3 in NOD mice.

Data are shown as mean±SEM, or as median±95% confidence interval where indicated. T: Total; D: Duodenal; S: Splenic.

Figure S3. β-cell distribution and innervation in STZ-treated mice.

A) Correlation between insulin+ islet number (left axis), β-cell volume (right axis) and blood glucose in control or STZ-treated mice. Islet volume, total: p=0.0055, duodenal: p=0.0012, splenic: p=0.0187; Islets per mm^3, total: p=0.0258, duodenal: p=0.0091, splenic: p=0.823, linear regression. N=18.

B) Correlation between exocrine nerve volume and blood glucose across in control or STZ-treated mice. Total: p=0.0719, duodenal: p=0.3879, splenic: p=0.4624, linear regression. N=15.

C) Correlation between blood glucose and endocrine nerve volume corrected for islet volume (left axis) or endocrine nerve volume per insulin+ islet (right axis) in control or STZ-treated mice. Nerve V / Islet V, total: p=0.0149, duodenal: p=0.6467, splenic: p=0.1547; Nerve V per islet, total: p=0.0396, duodenal: p=0.3790, splenic: p=0.200, linear regression. N=15.

D) Insulin+ islet volume distribution (left axis) and median insulin+ islet volume (right axis) in control or STZ-treated mice. Kruskal-Wallis test: p<0.001; Dunn's test: Control T *vs*. STZ day 15 T: p<0.001; Control D *vs*. STZ day 5 D, p=0.0027; Control D *vs*. STZ day 15 D, p<0.001; STZ day 5 T *vs*. STZ day 15 T, p<0.001; STZ day 5 D *vs*. STZ day 5 S, p<0.0020; STZ day 5 S *vs*. STZ day 15 S, p<0.001; STZ day 15 D *vs*. STZ day 15 S, p<0.001. Islets/group: 10479, 4682, 5797, 10091, 5162, 4929, 14380, 7543 and 6837.

E) Mean volume of insulin+ islets located <1.6 µm or >1.6 µm from the nearest nerve in control or STZ-treated mice. Kruskal-Wallis test: p<0.001. For islets <1.6 µm from innervation: Dunn's test: Control T *vs*. STZ day 15 T; p<0.001; Control D *vs*. Control S, STZ day 5 D, STZ say 15 D, p<0.001; STZ day 5 T *vs*. STZ day 15 T, p< 0.001; STZ day 5 S *vs*. STZ day 15 S, p<0.001; STZ day 15 D *vs*. STZ day 15 S, p<0.001. For islets >1.6 µm from innervation: Control T *vs*. STZ day 5 T, p<0.001; Control D *vs*. STZ day 5 D, p<0.001; STZ day 5 T *vs*. STZ day 15 T, p<0.001; STZ day 5 D *vs*. STZ day 5 S, p<0.001; STZ day 5 S *vs*. STZ day 15 S, p<0.001; STZ day 15 D *vs*. STZ day 15 S, p<0.001. Between STZ day 5 T, D and S: p<0.001. Between STZ day 15 T, D and S: p<0.001. Control T *vs*. STZ day 5 T, between islets <1.6 µm and >1.6 µm: p<0.001 for Control, STZ day 5 and STZ day 15 respectively, denoted by *** on bars. Islets/group: 237, 6384, 271, 4355, 508, 5680, 217, 3979, 145, 2614, 362, 3789, 307, 7233, 512, 5548, 819, 5395.

F) Proportion of innervated (NF200+) insulin+ islets with volumes <10,000 $µm^3$, 10,000–49,999 $µm^3$, 50,000–500,000 $µm^3$ or >50,000 $µm^3$ in control or STZ-treated mice.

Data are shown as mean±SEM, or as median±95% confidence interval where indicated. T: Total; D: Duodenal; S: Splenic.

Figure S4. Neuronal, endocrine and vascular markers in cleared pancreatic mouse tissue.

Maximum projections of small pancreatic samples cleared with iDISCO+ and immunolabeled for insulin with

A) NF200

B) Sympathetic marker, tyrosine hydroxylase (TH)

C) Parasympathetic marker, vesicular acetylcholine transporter (VAChT)

D) Sensory marker, Transient receptor potential cation channel subfamily V member 1 (TRPV1)

E) Pan-neuronal marker, synapsin

F) Glucagon

G) Somatostatin (SST)

H) Exocrine pancreas marker, mucin 1 (Muc1)

Maximum projections of small pancreatic samples cleared with a modified protocol for ECi and immmunolabeled for insulin and vasculature using:

I) Alexa-488 dextran

J) Alexa-647 CD31

Scale bars: 50 μm.

Figure S1

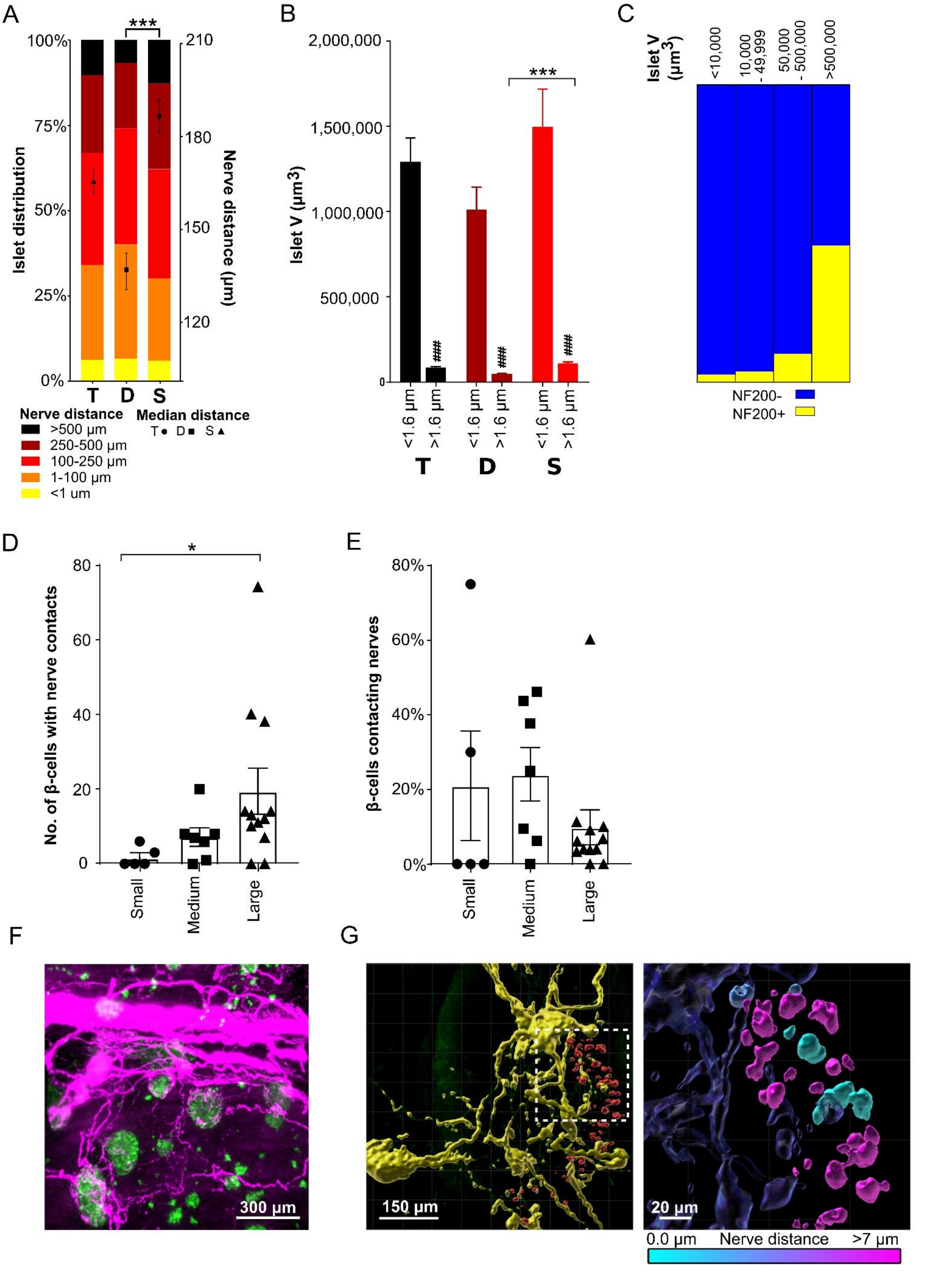
A
***
100%
75%
50%
25%
0%
210
180
150
120
Islet distribution
Nerve distance (µm)
T D S
Nerve distance
>500 µm
250-500 µm
100-250 µm
1-100 µm
<1 um
Median distance
T● D■ S▲
B
2,000,000
1,500,000
1,000,000
500,000
0
Islet V (µm3)
***
###
<1.6 µm
>1.6 µm
T D S
C
Islet V (µm3)
<10,000
10,000 - 49,999
50,000 - 500,000
>500,000
NF200-
NF200+
D
*
80
60
40
20
0
No. of β-cells with nerve contacts
Small
Medium
Large
E
80%
60%
40%
20%
0%
β-cells contacting nerves
Small
Medium
Large
F
300 µm
G
150 µm
20 µm
0.0 µm
Nerve distance
>7 µm

Figure S2

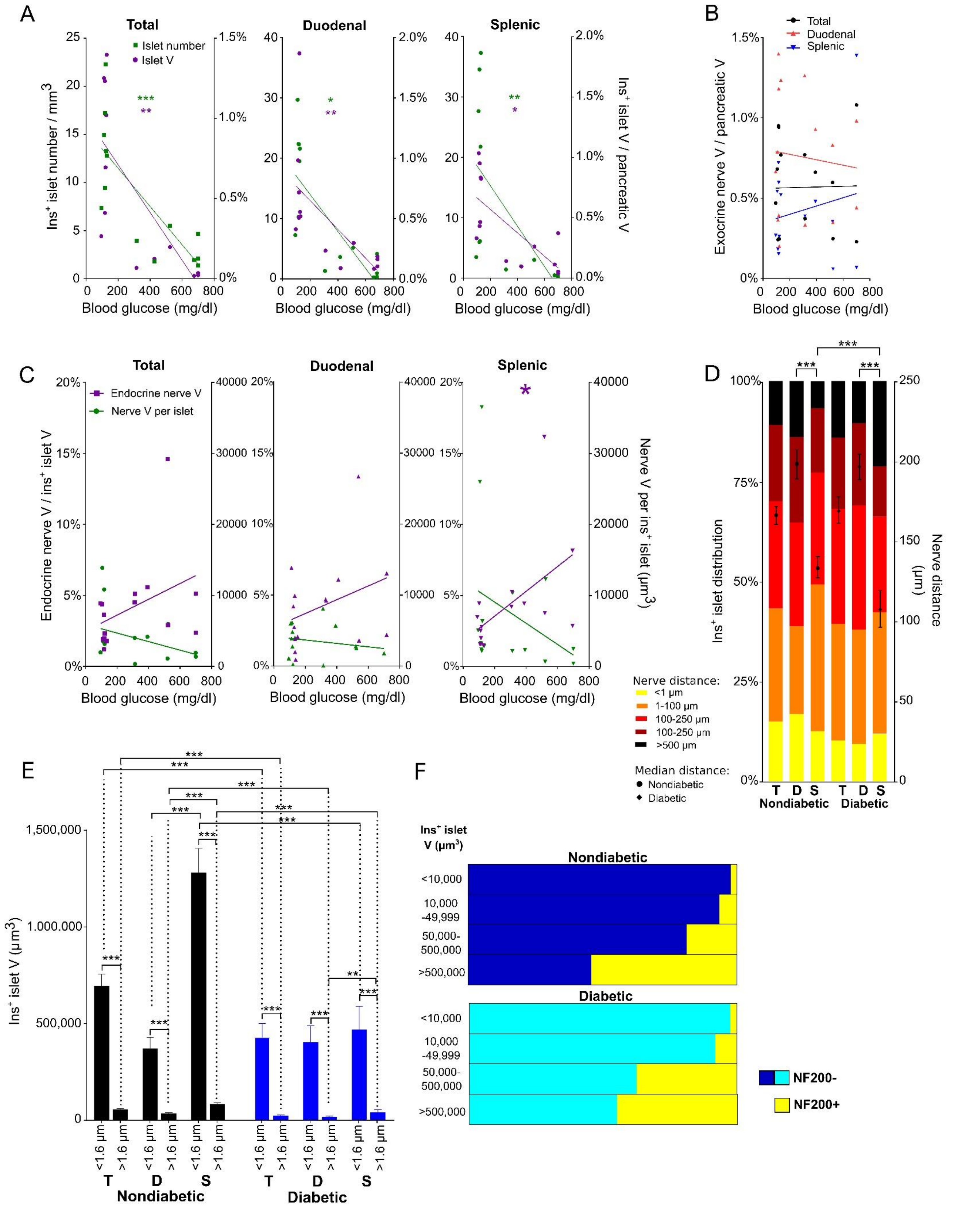

A
Total
Duodenal
Splenic
Islet number
Islet V
Ins+ islet number / mm3
Ins+ islet V / pancreatic V
Blood glucose (mg/dl)
B
Total
Duodenal
Splenic
Exocrine nerve V / pancreatic V
C
Endocrine nerve V
Nerve V per islet
Endocrine nerve V / ins+ islet V
Nerve V per ins+ islet (µm3)
D
Ins+ islet distribution
Nerve distance (µm)
Nerve distance:
<1 µm
1-100 µm
100-250 µm
100-250 µm
>500 µm
Median distance:
Nondiabetic
Diabetic
T D S T D S
Nondiabetic Diabetic
E
Ins+ islet V (µm3)
<1.6 µm
>1.6 µm
Nondiabetic
Diabetic
F
Ins+ islet V (µm3)
Nondiabetic
Diabetic
<10,000
10,000 -49,999
50,000- 500,000
>500,000
NF200-
NF200+

Figure S3

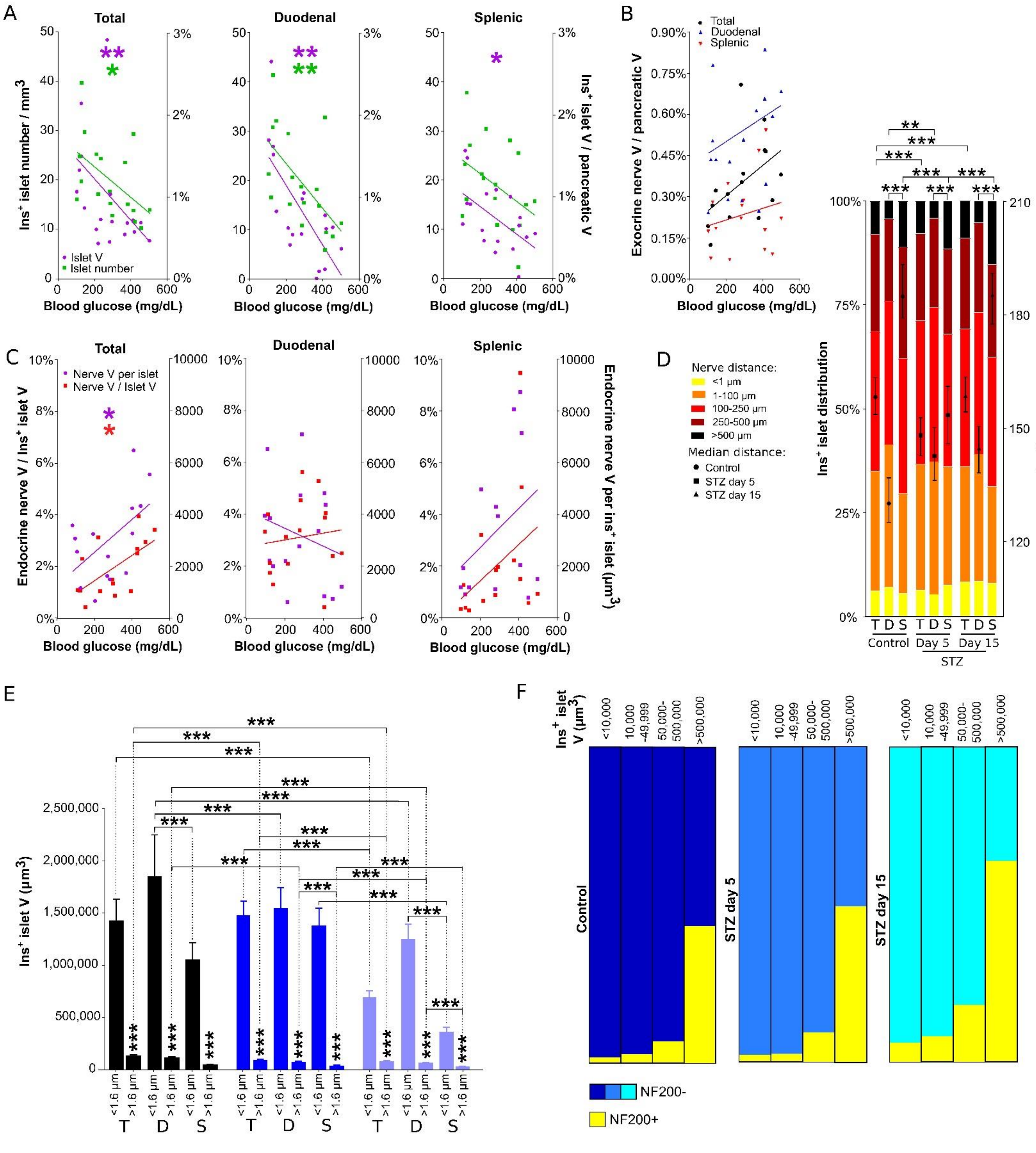
A
Total
Duodenal
Splenic
Ins+ islet number / mm3
Ins+ islet V / pancreatic V
Islet V
Islet number
Blood glucose (mg/dL)
B
Total
Duodenal
Splenic
Exocrine nerve V / pancreatic V
Blood glucose (mg/dL)
C
Total
Duodenal
Splenic
Nerve V per islet
Nerve V / Islet V
Endocrine nerve V / Ins+ islet V
Endocrine nerve V per ins+ islet (µm3)
Blood glucose (mg/dL)
D
Nerve distance:
<1 µm
1-100 µm
100-250 µm
250-500 µm
>500 µm
Median distance:
Control
STZ day 5
STZ day 15
Ins+ islet distribution
Nerve distance (µm)
T D S
Control
Day 5
Day 15
STZ
E
Ins+ islet V (µm3)
<1.6 µm
>1.6 µm
T
D
S
F
Ins+ islet V (µm3)
<10,000
10,000 -49,999
50,000-500,000
>500,000
Control
STZ day 5
STZ day 15
NF200-
NF200+

Figure S4

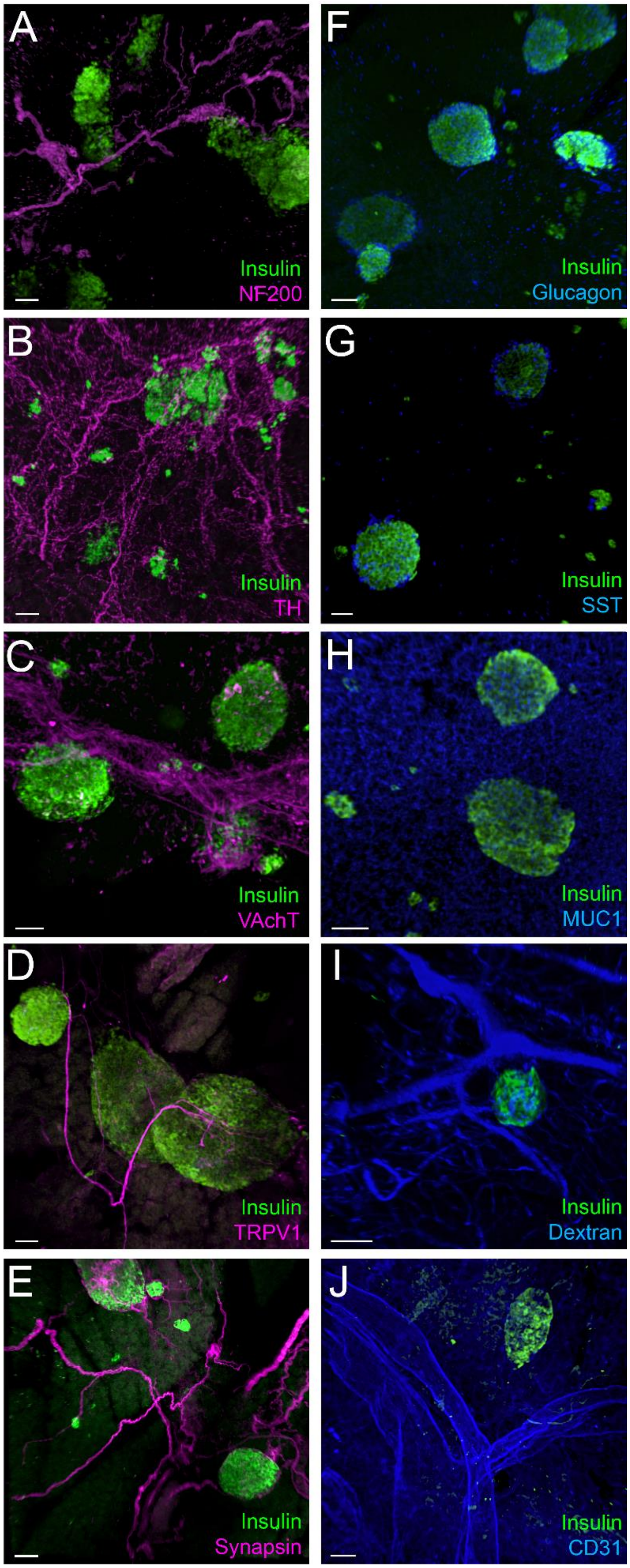
A
Insulin
NF200
B
Insulin
TH
C
Insulin
VAchT
D
Insulin
TRPV1
E
Insulin
Synapsin
F
Insulin
Glucagon
G
Insulin
SST
H
Insulin
MUC1
I
Insulin
Dextran
J
Insulin
CD31

Table S1. List of evaluated antibodies.

**Primary antibodies**						
**Target**		**Dilution**	**Host species**	**Manufacturer**	**Product number**	**RRID number**
Insulin		1:1000	Guinea pig	Dako / Agilent	A0564	AB_10013624
Neurofilament 200 kDA		1:500	Rabbit	Sigma-Aldrich	N4142	AB_477272
Tyrosine Hydroxylase		1:500	Rabbit	Millipore	AB152	AB_390204
Vesicular Acetylcholine Transporter		1:500	Rabbit	Synaptic Systems	139 103	AB_887864
Glucagon		1:200	Rabbit	Cell Signaling Technology	2760	AB_659831
Glucagon		1:2000	Mouse	Sigma-Aldrich	G2654	AB_259852
Somatostatin		1:1000	Goat	Santa Cruz	SC7819	AB_2302603
Mucin 1		1:200	Armenian hamster	Thermo Fisher Scientific	MA5-11202	AB_11000874
Transient receptor potential cation channel subfamily V member 1		1:500	Rabbit	Alomone labs	ACC-030	AB_2313819
Synapsin		1:500	Rabbit	Cell Signaling Technology	5297	AB_2616578
Insulin		1:500	Rat	R&D Systems	MAB1417	AB_2126533
CD31		50 mg/ml	Rat	Biolegend	102515	AB_2161030
**Secondary antibodies**						
**Target**	**Conjugate**	**Dilution**	**Host species**	**Manufacturer**	**Product number**	**RRID number**
Guinea pig	Alexa Fluor® 647	1:500	Donkey	Jackson ImmunoResearch	706-605-148	AB_2340476
Rabbit	Alexa Fluor® 647	1:500	Donkey	Jackson ImmunoResearch	711-605-152	AB_2492288
Rabbit	Alexa Fluor® 594	1:500	Donkey	Invitrogen	A-21207	AB_141637
Rabbit	Alexa Fluor® 546	1:500	Donkey	Thermo Fisher Scientific	A10040	AB_2534016
Mouse	Alexa Fluor® Plus 647	1:500	Donkey	Invitrogen	A32787	AB_2762830
Goat	Alexa Fluor® 546	1:500	Donkey	Thermo Fisher Scientific	A-11056	AB_2534103
Armenian Hamster	Alexa Fluor® 594	1:500	Goat	Jackson ImmunoResearch	127-585-099	AB_2338998
Rat	DyLight® 550	1:500	Donkey	Thermo Fisher Scientific	SA5-10027	AB_2556607
Rat	Alexa Fluor® 488	1:500	Donkey	Thermo Fisher Scientific	A-21208	AB_25357794
